# The application of *Rumex abyssinicus* based activated carbon for Brilliant Blue Reactive dye adsorption from aqueous solution

**DOI:** 10.1186/s13065-023-01004-2

**Published:** 2023-07-18

**Authors:** Ashagrie Mengistu, Mikiyas Abewaa, Eba Adino, Ebisa Gizachew, Jemal Abdu

**Affiliations:** 1 The Federal Democratic Republic of Ethiopia, Manufacturing Industry Development Institute, P. O. BOX 1180, Addis Ababa, Ethiopia; 2 Department of Chemical Engineering, College of Engineering and Technology, Wachemo University, P. O. Box 667, Hossana, Ethiopia; 3 Department of Chemical Engineering, School of Mechanical, Chemical and Materials Engineering, Adama Science and Technology University, P.O. Box 1888, Adama, Ethiopia

**Keywords:** Adsorbent, Characterization, Brilliant blue, *Rumex abyssinicus*, Wastewater

## Abstract

The environmental pollution and human health impacts associated with the discharge of massive dye-containing effluents necessitate a search for cost-effective treatment technology. Therefore, this research work is conducted with the objective of investigating the potential of *Rumex abyssinicus*-derived activated carbon (RAAC) for the adsorption of Brilliant Blue Reactive (BBR) dye from aqueous solutions. Chemical activation with H_3_PO_4_ followed by pyrolysis was used to prepare the adsorbent. Characterization of the developed adsorbent was done using proximate analysis, pH point of zero charge (pHpzc), scanning electron microscope (SEM), Fourier transform infrared spectrometer (FTIR), Brunauer, Emmett, and Teller (BET), and X-ray diffraction (XRD). The experimental design and the effect of independent variables including pH (2, 6, and 10), initial dye concentration (50, 100, and 150 mg/L), adsorbent dosage (0.05, 0.1, and 0.15 g/100 mL), and contact time (20, 50, and 80 min) were optimized using the response surface methodology (RSM) coupled with Box Behnken design (BBD). The analysis results revealed the exitance of high specific surface area of 524 m^2^/g, morphological cracks, and the presence of multiple functional groups like –OH, C=C, alkene, and amorphous structure. Maximum removal efficiency of 99.98% was attained at optimum working conditions of pH 2, contact time of 50 min, dye concentration of 100 mg/L, and adsorbent dosage of 0.15 mg/100 mL, reducing the pollutant concentration from 100 to 0.02 mg/L. Evaluation of the experimental data was done using Langmuir, Freundlich, Temkin, and Sips isotherm models, in which the Langmuir model was found to be the best fit with the experimental data at R^2^ 0.986. This shows that the adsorbent surface is homogeneous and mono-layered. Furthermore, the kinetic study confirmed that the pseudo second-order model best describes the experimental data with R^2^ = 0.999. In general, the research work showed that the low cost, environmental friendliness and high adsorption capabilities of the activated carbon derived from *Rumex abyssinicus* could be taken as an effective nt for the removal of BBR dye from aqueous solutions.

## Introduction

A water resource refers to natural sources of water that include rivers, lakes, springs, oceans, seas, and others. These resources are considered crucial for humans and other living beings in one way or another. Water use can typically range from drinking purposes and irrigation to industrial applications as either as a process liquid or utility liquid. Water resources can be affected by rapid climate changes, such as changes in temperature and precipitation [1–3]. These and other factors may lead to water scarcity, a term that refers to the mismatch between water supply and demand in society or other ecosystems. Basically, water scarcity refers to the availability of freshwater below 1000 L per person per year, and it is estimated that 40% of the world's population will end up water scarcity by 2030. In line with water scarcity, 4.8–5.7 billion people will be at risk by 2050 [4–6]. This needs a call not only for an action for efficient allocation of water resources but also for the potential reuse of wastewater streams from different industrial setups. Wastewater is any used water originating from industries, households, residential areas, etc. and whose biological, chemical, and physical properties have been altered. The alteration of any of these water properties has a deleterious effect on public and environmental health and is usually called water pollution [1]. Sources of the pollution could be anthropogenic activity or natural activity; industrial discharges, dumping waste material into water streams, and other economic activities are some of the human-made sources of water pollution, while the natural source is mainly associated with geological activities within the earth [2, 3, 7–9]. Water pollution can cause detrimental health issues for humans, where the root causes of diseases such as cancer, diarrhea, and other waterborne diseases are usually traced back to water pollution [10, 11]. Therefore, protecting the quality of water, which is diminishing, is becoming one of the top agendas of the scientific community.

Currently, more than 100,000 commercial dyes are available. Of these, 1000 dyes are being used in different stages of textile processing, which makes the estimated annual production rate of dyes to be 700,000 tonnes [12]. It was reported that about 15% of the dyes used in industry are eventually released into the environment after being applied in a dyeing process [13]. Methyl orange (MO), Rhodamine B (RhB), Methylene Blue (MB), Congo red (CR), Brilliant Blue Reactive (BBR), and Reactive Black-5 (RB-5) are among the widely used dyes in the textile industries [14], which are classified into anionic, neutral, and cationic dyes [15]. BBR is an anionic dye mostly in use in textile dyeing and finishing processes and is well known to have significant impact on the environment and human health if existed at high concentration in industrial effluents or aquatic environments [16]. This toxic dye was reported to be present in the textile wastewater with a concentration ranging from 50 to 150 mg/L [17]. BBR is a carcinogenic and mutagenic compound that poses a detrimental impact on human health [18]. Furthermore, the massive concentration of the BBR dye in the wastewater that was later discharged into the nearby water bodies limits the penetration of sunlight into the bottom part of the river, which in turn disturbs the aquatic ecosystem [19]. Therefore, treatment intervention is critical not only for aesthetics but also to protect aquatic biota.

Conventional wastewater treatment plants are ineffective at removing micropollutants from industrial effluent. In practice, they have failed to decrease dye concentrations, particularly reactive dyes, to acceptable discharge limits. Besides this, they also perform at well-timed operations and require a higher cost of implementation [20]. A number of studies have revealed that advanced wastewater treatment methods can help solve this problem [4, 21]. Chemical precipitation, nanofiltration, advanced oxidation, ion exchange, reverse osmosis, membrane separation, electrocoagulation, and electrodialysis are the most common advanced wastewater treatment technologies applied for the removal of dyes from aqueous solutions, and their effectiveness has been thoroughly studied. For instance, photocatalytic degradation of methyl orange via CuAl_2_O_4_ nanoparticles [22] and membrane filtration of dyes [23] are well known advanced waste water treatment technologies being in use especially in developed countries. In line with this, the removal of BBR dye has become the subject of current researches, where various studies employing different mechanisms using advanced technologies have been conducted to decontaminate BBR dye from aqueous solutions [24–26]. However, these technologies are a very expensive and non-sustainable wastewater treatment mechanism to have them in every sectors, particularly those in developing nations [27]. Adsorption is a process that is proven to be cost effective, convenient, easy to develop, and environmentally beneficial [28]. Activated carbon among adsorbents is increasingly being regarded as the best alternative to remove toxic dye from water and wastewater [29]. It is a porous carbonenous material that has a great attraction for pollutants because of its porous structure, surface multifunctionalities, and large specific surface area [30] and its application as a wastewater treatment technology like dye decolorization has been studied thoroughly. For instance, the application of activated carbon derived from pomegranate peels for the adsorption of basic red 46 [31], and the adsorptive removal of cationic dye onto activated carbon derived from cactus fruit peels [32] are some of the important examples of application of activated carbon. In addition to this, various adsorbent materials are applied for the removal of BBR dye [23, 33, 34].

Nowadays, the search for alternative raw materials for the adsorption of toxic and persistent dyes such as BBR is increasing. This is due to the non-sustainability and cost of commercial activated carbon. Thus, various biomass-based activated carbons are being studied and addressed for their effectiveness in removing pollutants by a number of scholars [5, 6, 27, 35–37]. However, these studies have their own drawbacks of low adsorption capacity, consuming a large amount of adsorbent for a minute quantity of pollutant detoxification, performing well only at a longer contact time, and having low removal efficiency. Therefore, *Rumex abyssinicus* derived adsorbent material is selected to fill gaps like low specific surface areas and the minimum adsorption capacities of various adsorbents. *Rumex abyssinicus* is an herb that is mainly distributed throughout tropical Africa, mostly in Ethiopia [38]. It is a plant that is 3–4 m tall [39]. So far, the root of *Rumex abyssinicus* has been utilized for medicinal purposes [40, 41]. However, the remaining parts of the plant, such as the stem and leaves, are usually thrown away as waste. Moreover, to the best of our knowledge, the application of the stem of *Rumex abyssinicus* for environmental clean-up has not been done before and no research has been reported on the removal of BBR dye from aqueous solutions and wastewater using activated carbon synthesized from the stem of *Rumex abyssinicus*, which is a waste of precious raw material. Therefore, this research work is conducted with the objective of preparing activated carbon from *Rumex abyssinicus* and evaluates its performance in removing BBR from aqueous solutions. Moreover, characterization of the developed activated carbon, data fitness analysis on kinetics and isotherms models, regeneration and reusability and cost analysis to check production feasibility are among the specific objectives of this research work. The Box–Behnken approach to response surface methodology was used to optimize batch adsorption, consisting of four factors. These factors with their levels are pH (2, 6, and 10), initial dye concentration (50, 100, and 150 mg/L), adsorbent dosage (0.05, 0.1, and 0.15 g/100 mL), and contact time (20, 50, and 80 min).

## Materials and methods

### Chemicals and materials

The precursor biomass (*Rumex abyssinicus*) was collected from the Addis Ababa Science and Technology University premise, Addis Ababa, Ethiopia. Analytical-grade chemicals and reagents such as sodium hydroxide pellets (99.0%), hydrochloric acid (37% w/w), phosphoric acid (88%), and BBR dye were used for laboratory experiments. Furthermore, digital electronic balance (Shimazu-AUX220), digital pH meter (PHS-3C), coffee grinder (model: XFYC810, 240 V, 60 Hz), orbital shaker (model: SK-600, 230VAC, 50 Hz, 0.2A by QTEX), UV/VIS spectrometer (Evolution 300, UV–VIS by Maalab), hot air oven (model: J-dvo1, supplier: JISICO), muffle furnace (Carbolite AAF 1100), testing equipment such as Fourier transform infrared spectroscopy (FT-IR), IR Affinity, Shimadzu), x-ray power diffraction (XRD-X-ray tube cu40kV, 44 mA, Rigaku, Ultima IV), and scanning electron microscope (model JSM840A SEM microscope operating at 10 kV) were the equipment employed during the study. Finally, the main properties of the target pollutant (BBR dye) are presented in Table 1.

**Table 1:** Main properties of Brilliant Blue Reactive dye

Pollutant name	Brilliant Blue reactive
Molecular weight	825.972 g/mol
Class	Anionic

### Adsorbent preparations

Sun-dried stems of *Rumex abyssinicus* were collected from the Addis Ababa Science and Technology University premise which is found in Addis Ababa, Ethiopia. The voucher plant specimen (with no ID) was deposited in a university herbarium (Addis Ababa University) and the plant specimen was collected by the researchers (Ashagrie Mengistu and Mikiyas Abewaa) and crossed checked against the herbarium. The plant identification was performed by an expert assigned to the herbarium site. The collected sample was then size reduced using a knife, i.e., 5–10 mm, washed thoroughly with tap water, rinsed with distilled water, and dried in an oven at 105 °C for 24 h. Thereafter, the oven-dried sample of *Rumex abyssinicus* was impregnated with phosphoric acid (88%), after dilution to a 50% solution, using a mass-to-mass ratio of 1:2, and then allowed it to soak for 8 h at room temperature. Then, the impregnated sample was air-dried in an oven at 105 °C for 24 h, to remove excessive water and create a conducive environment for the successful carbonization of the sample. The pyrolysis process took place at a thermal activation temperature of 500 °C and a carbonization time of 2 h with in a glowing red furnace. Finally, the thermally activated sample was taken out of the furnace, allowed to cool in the desiccator, and repeatedly washed with distilled water until the pH of the adsorbent had reached neutral. Finally, after being dried in the oven, the adsorbent material was ground to a particle size of 250 μm and stored in the airtight plastic container for subsequent experiments of adsorbent characterization, batch adsorption, kinetics and isotherm model fitness studies [5, 27, 35].

### Adsorbent characterization

The developed *Rumex abyssinicus*-derived activated carbon was subjected to proximate analysis, which included measuring its moisture content, volatile matter, ash content, and fixed carbon in accordance with the ASTM protocols (D2866–2869), respectively. On the other hand, the pH point of zero charge (pHpzc) was determined using the mass titration method, where 0.1 M of 50 mL NaCl is prepared using six 250 mL Erlenmeyer flasks the pH of which were adjusted to 2, 4, 6, 8, 10, and 12, respectively using either 0.1 M NaOH or 0.1 HCl. After adding an equal amount i.e., 1 g of the sample and shaking on orbital shaker for 24 h, the final pH for all solutions were assessed. Then, initial pH and final pH values were used to draw the curve and the intersection of the two lines was then taken as pHpzc. The specific surface area and functional group analysis of the produced adsorbent were carried out using BET (model SA-9600 series from Japan) and FTIR (Perklin Elmer, USA), respectively. Where, FTIR scanning was performed on the wavenumber range of 4000–400 cm^−1^ after the sample was properly mixed with KBr. On the other hand, the surface morphology and crystalline structure of the activated carbon was examined using SEM and XRD (INSPECT, F50, USA, and XRD-7000, Japan), respectively [42–44].

### Box Behnken experimental design

Design expert software was used to carry out the experimental design and the adsorption optimization process using response surface methodology under the Box–Behnken approach. The design consists of four independent variables, namely pH, contact time, adsorbent dosage, and initial dye concentration, all with three levels. However, the dependent variable (the response variable) is removal efficiency. The levels for the experimental factors were symbolized as low (−), middle (0), and high (+). Normally, the full factorial design of four factors at three levels (3^4^) is anticipated to generate 81 experimental runs. However, the Box–Behnken approach to response surface methodology reduces the number of batch experimental runs to 30. Table 2 presents the independent variables and their corresponding levels selected based on the preliminary studies done and related published articles from the literature. Finally, the interaction effect of various independent variables on the dye removal as well as the statistical analysis of the response variable (predicted vs. actual) were carried out with the aid of ANOVA and regression modelling.

**Table 2:** Independent variables and their corresponding levels

Variables	Units	Low (–)	Middle (0)	High (+)
pH	–	2	6	10
Dye concentration	mg/L	50	100	150
Adsorbent dosage	g/100 mL	0.05	0.1	0.15
Contact time	min	20	50	80

### Batch adsorption experiment

**Fig. 1:**
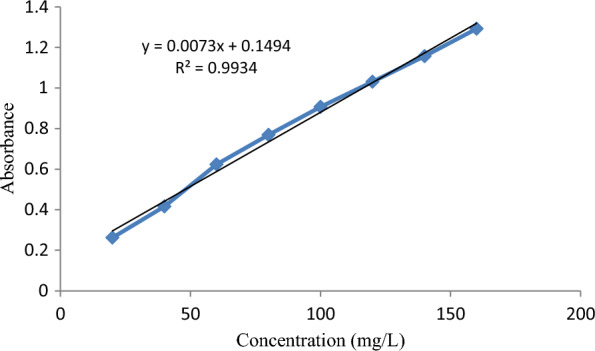
Calibration curve development to determine unknown concentration for BBR dye

The batch adsorption experiments were carried out using the already designed experiments, where the three levels of concentration (50, 100, and 150 mg/L) for the adsorbate were prepared by dissolving 50, 100, and 150 mg of BBR dye each in 1 L of distilled water, respectively. A working volume of 100 mL with a suitable concentration was added to several Erlenmeyer flasks of 250 mL. Then, the pH of the solution was adjusted using either 0.1 M NaOH or 0.1 M HCl, depending on the requirement. Thereafter, the known amount of *Rumex abyssinicus*-based activated carbon was added to the solution based on the design experiment. The mixture was allowed to be agitated on the orbital shaker at 125 rpm for the specified duration of contact time. As the contact time was completed, the solution was filtered using Whatman filter paper 42, and the filtrate was taken to determine the amount of dye concentration remaining in the solution. The final dye concentration was determined by using a UV–VIS spectrophotometer (Agilent Technology, Cary 100 UV–visible spectrophotometer) at a maximum wavelength of 593. For this purpose, a calibration curve was drawn using 20, 40, 60, 80, 100, 120, 140, and 160 mg/L of BBR dye. The coefficient of determinant R^2^ of 0.99 was determined from the linear plot of dye concentration against absorbance as shown in Fig. 1. Normally, an R^2^ approaching 1 indicates the data best fits the linear plot, which in turn is used to determine the unknown dye concentration. The linear equation generated from the plot was used for the determination of the unknown BBR concentrations. Then, the final concentrations of the 30 experimental runs were determined indirectly by using the Beer–Lambert equation. Finally, the adsorption capacity and removal efficiency of BBR dye were determined using Eqs. 1 and 2, respectively.

1 $$ Q_{t} = \left( {\frac{{C_{o} - C_{t} }}{m}} \right) \times V, $$

2 $$ {\text{Re }}\left( \% \right) = \frac{{C_{O} - C_{e} }}{{C_{O} }} \times 100, $$

where Q_t_ (mg/g) is adsorption capacity at time t, however Co and C_t_ (mg/L) represent the initial and dye concentration at t respectively. On the other hand, R_e_ represents removal efficiency, m and V refers to mass of the adsorbent and volume of the solution respectively [4–6, 35].

### Adsorption isotherm

Langmuir, Freundlich, Temkin, and Sips, models are the well-known adsorption isotherms used to evaluate the nature of interaction between the adsorbate and adsorbent at equilibrium. These models are presented in Table 3. With this regard, the interaction is said to be monolayer if the data fits well to the Langmuir isotherm and multilayer if the data fit is inclined to the Freundlich model. Moreover, the surface interaction is heterogeneous if the data is well represented by Freundlich and homogenous if the Langmuir model is found to be explanatory. On the other hand, Temkin isotherm gives a clue to heat transfer during adsorption. The Sips isotherm, which is a combination of Langmuir and Freundlich models, is used for predicting the adsorption on heterogeneous surfaces. The adsorption isotherm experiment was conducted at fixed optimum values of pH 2, adsorbent dosage of 0.15 g/100 mL, and a contact time of 50 min, whereas the initial dye concentration varied from 40 to 140 mg/L (40, 60, 80, 100, 120, and 140 mg/L).

**Table 3:** Adsorption isotherm models with linear and non- linear forms

Isotherm model	Non-linear form	Linear form	References
Langmuir	${\text{Qe}} = \frac{QmaxKLCe}{{1 + KLCe}}$	$\frac{1}{Qe} = \frac{1}{Qmax} + \frac{1}{KLQmaxCe}$	[45–47]
Freundlich	Qe = KFCe^1/n^	Log Qe = log KF + $\frac{1}{n}$ log Ce	[46, 48, 49]
Temkin	Qe = $\frac{RT}{b}$ ln($K_{T}$ Ce)	Qe = $\frac{RT}{b}lnCe + \frac{RT}{b}lnK_{T}$	[46, 48, 50]
Sips	Qe = $$\frac{{Qmax(KCe)^{n} }}{{(1 + KCe)^{n} }}$$	ln $$\left( {{\raise0.7ex\hbox{${Qe}$} \!\mathord{\left/ {\vphantom {{Qe} {Qmax}}}\right.\kern-0pt} \!\lower0.7ex\hbox{${Qmax}$}} - Qe} \right)$$ = $${\raise0.7ex\hbox{$1$} \!\mathord{\left/ {\vphantom {1 {ns}}}\right.\kern-0pt} \!\lower0.7ex\hbox{${ns}$}}$$ ln Ce + ln $$(Ks)^{ns}$$	[32, 45]

Additionally, the adsorption dimensionless factor constant $\left( {R_{L} } \right)$ that is used to estimate Langmuir isothermal feasibility is shown in Eq. 3.

3 $$ {\text{RL}} = \frac{1}{{1 + {\text{K}}_{{\text{L}}} {\text{Ce}}}}, $$

where q_max_ (mg/g) is maximum monolayer adsorption capacity, K_L_ (L/mg) is Langmuir constant, which is related to free energy of the adsorption, $R_{L}$ is affinity between adsorbate and adsorbent, Kf (mg/g) is Freundlich constant indicating adsorption capacity and 1/n is an empirical equation related to intensity of the adsorption. Moreover, $K_{T}$ and $K_{S}$ refer to Temkin and Sips isotherm constant respectively [51, 52].

### Adsorption kinetics

The mechanism of adsorption as well as the rate of adsorbate uptake was determined using adsorption kinetics models. The adsorption kinetics was evaluated using pseudo-first order (PFO), pseudo-second order (PSO), the Intraparticle diffusion model (IPD), and the Elovich models. These kinetic models are also used to determine the potential rate-determining step in the adsorption process. In this approach, pH, adsorbate concentration, and adsorbent dosage were fixed to optimum values of 2, 100 mg/L, and 0.15 g/100 mL, respectively, whereas the contact time was varied between 20 and 50 min (20, 30, 40, 50, and 60 min). Table 4 presents linear and non-linear equations of kinetics models applied for examining the best data fit, where $$ k_{1} ,$$ $$k_{2}$$ and $$k_{d}$$ are PFO, PSO, and IPD rate constants. Similarly, C is a constant that gives information about the boundary layer, α and β are the initial adsorption rate and desorption constant during each experiment, respectively [53–55].

**Table 4:** Linear and nonlinear forms of adoption kinetics models

Kinetics model	Non-linear equation	Linear equation	References
PFO	Qt = Qe(1 − $e^{ - K1t}$)	$\log \left( {Qe - {\text{Qt}}} \right)$ = $\log Qe$ − $\frac{K1t}{{2.303}}$	[47, 50]
PSO	Qt = $\frac{{Qe^{2} K_{2} t}}{{1 + QeK_{2} t}}$	$\frac{t}{Qt}$ = $\left( \frac{1}{Qe} \right)$t + $\frac{1}{{K2Qe^{2} }}$	[47, 50]
IPD	Qe = kd × t^0.5^ + C	Qe = kd × t^0.5^ + C	[32, 50]
Elovich	Qt = $\frac{1}{\beta }$ ln($\alpha \beta t + 1$)	Qt = $\frac{1}{\alpha }$ ln (αβ) + $\frac{1}{ \uparrow \alpha }$ ln t	[45, 56]

## Result and discussion

### Adsorbent characteristics

Proximate parameters (moisture content, ash content, volatile matter, and fixed carbon) of the adsorbent were used to determine the quality of the activated carbon prepared. Normally, an adsorbent with small amounts of ash content, moisture content, and volatile matter and those with large fixed carbon content are considered as a suitable precursor material for the adsorption technology. However, the proximal values of activated carbon are influenced by the treatment method used and the nature of the precursor material. The proximate analysis of RAAC was indicated in Table 5. It can be observed from the table that the moisture content of RAAC was determined to be 3.4%, which is lower than previously reported results of biomass-based activated carbons like, castor seed hull (4.4%) [43], fox nut (4.00%) [57], Paulownia wood (3.50%) [58], and tomato stems (3.58%) [59]. High moisture content adds mass to the activated carbon without contributing to adsorption hence reducing the share of the carbonous material. This condition forces us to utilize more adsorbent dosage to attain the desired degree of adsorption, which indirectly affects the adsorption capacity. In line with the effects of moisture on the adsorbent structures, it was reported that competition of the water molecule in the moisture with the adsorbate for the active site as well as filling adsorbent active sites will reduce the adsorption efficiency [60]. On the other hand, the volatile matter content of RAAC was found to be 16.2%, which is lower than many of the previously reported findings for biomass based adsorbents. For example, *Euphorbia rigida* (76.80%) [61], Acai seed (68.70%) [62], Paulownia wood (17.8%) [58], fox nut (21.5%) [57] and castor hull (49.56%). The lower volatile matter content of the RAAC is due to chemical activation. Normally, the volatile matter is related to the mass escaped (${\text{H}}_{2} ,$ CO, ${\text{CO}}_{2} ,$ ${\text{CH}}_{4} ,$ ${\text{N}}_{2}$ and Hydrocarbons) during pyrolysis and chemical activation processes. Similarly, the ash content and fixed carbon content of the RAAC were found to be 9.5 and 70.9% respectively. Ash content is associated with the inorganic constituents of activated carbon like metal oxides. These inorganic constituents of the activated carbon do not contribute to adsorption but rather block the voids to which the target pollutant is intended to be attached. RAAC’s ash content of the adsorbent material was compared with other biomass based adsorbents and the result was found to be promising. For instance [43] reported ash content of 19.1% for castor hull derived activated carbon and durian shells (22.36%) [63]. The fixed carbon content of the activated carbon indicated the quality of the adsorbent, and it was as expected to be high making the developed adsorbent promising. The adsorbent currently under investigation found to have fixed carbon content of 70.9%, which is an indicator of the effectiveness of the adsorbent at removing BBR dye from aqueous solution. This fixed carbon content is higher than castor seed hull derived activated carbon (16.89%) [43] and Durian shell (22.30%) [63] but slightly lower than fox nut (73.1%) [57]. Generally, the proximate values of RAAC are in agreement with standard activated carbon reported by [64].

**Fig. 2:**
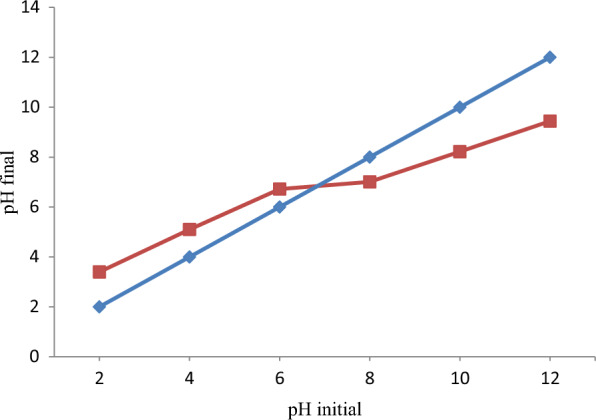
pHpzc analysis of *Rumex abyssinicus* based activated carbon

pHpzc is the value of pH at which the components of surface charge are equal to zero for a particular temperature, pressure, and aqueous solution components. At pH equal to pHpzc, there are equal amounts of negative and positive charges. pHpzc of RAAC was determined through the mass addition method and the result of the analysis is depicted in Fig. 2. Normally, pHpzc determination is intended to investigate the surface density of the adsorbent. At pH = pHpzc the surface density of the adsorbent becomes neutral indicating an equal amount of positive and negative charges. At the pH of the adsorbent below its pHpzc positive charges dominates the surface and above pHpzc the surface of the adsorbent will be dominated by negative charge. Hence, the adsorption of anionic dyes like BBR usually favours at a pH of the solution below pHpzc whereas cationic dye adsorption is favoured at pH greater than pHpzc. The pHpzc of RAAC is determined to be 6.9. Hence, maximum adsorption of BBR dye is expected at a pH of solution less than 6.9. Herein, the maximum removal efficiency of 99.98% recorded at pH = 2 confirms the pHpzc concept. Finally, the currently determined pHpzc of RAAC is in good agreement with [65, 66].

**Table 5:** Proximate analysis of RAAC applied for removal of BBR dye

Proximate analysis	Values (%)
Moisture content	3.4 ± 0.23
Volatile matter	16.2 ± 1.02
Ash content	9.5 ± 0.44
Fixed carbon	70.9 ± 1.69

**Fig. 3:**
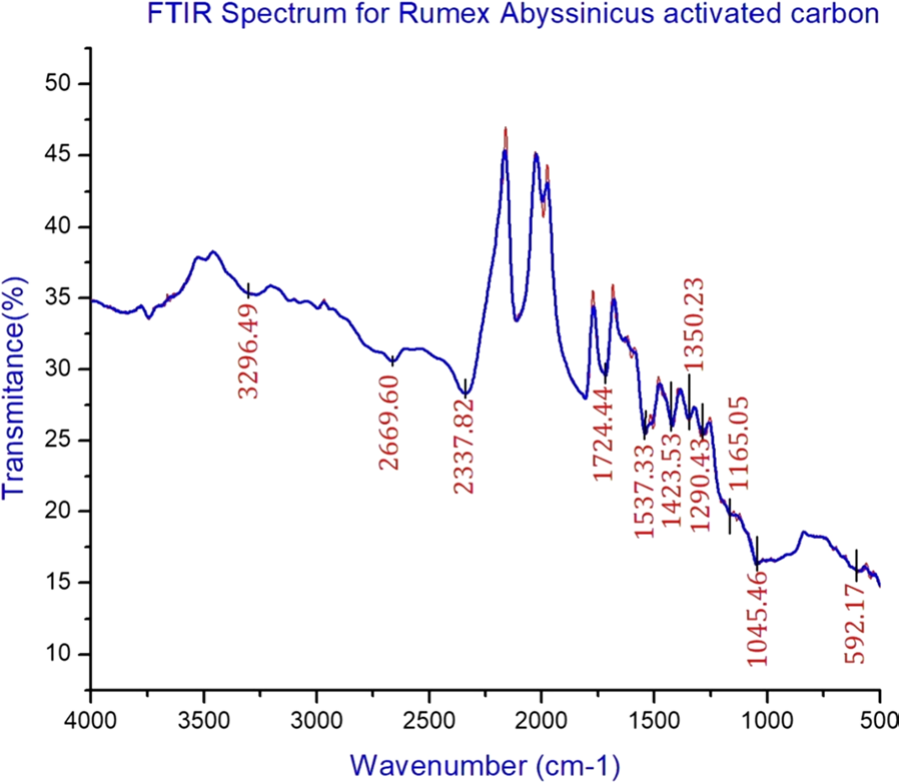
Functional group analysis of RAAC

Functional group analysis of *Rumex abyssinicus* derived activated carbon was carried out using FTIR and the result of the finding is shown in Fig. 3. The FTIR analysis of RAAC resulted in several peaks showing the presence of multiple functional groups in the adsorbent. These peaks are observed at wavenumbers of 3296.49, 2669.60, 2337.82, 1724.44, 1537.33, 1423.53, 1350.23, 1290.43, 1165.05, 1045.46 and 592.17 cm^−1^. The peak observed at 3296.49 cm^−1^ is attributed to –OH—containing functional groups like water and alcohol. The relatively less intense peak observed in the region of 1500–1210 cm^−1^ corresponds to the stretching motions of –C–H groups. On the other hand, alkene groups are represented by the peak observed at 592.17 cm^−1^. The intense peak observed at 1537.33 cm^−1^ is attributed to C=O whereas, the shoulder band indicated in the wavelength spectrum of 2669.60 cm^−1^ is attributed to –CH_2_ and –CH_3._ On the other hand, C=O vibration is indicated by the spectrum observed at 1724.44 cm^−1^ [4–6, 27, 35].

**Fig. 4:**
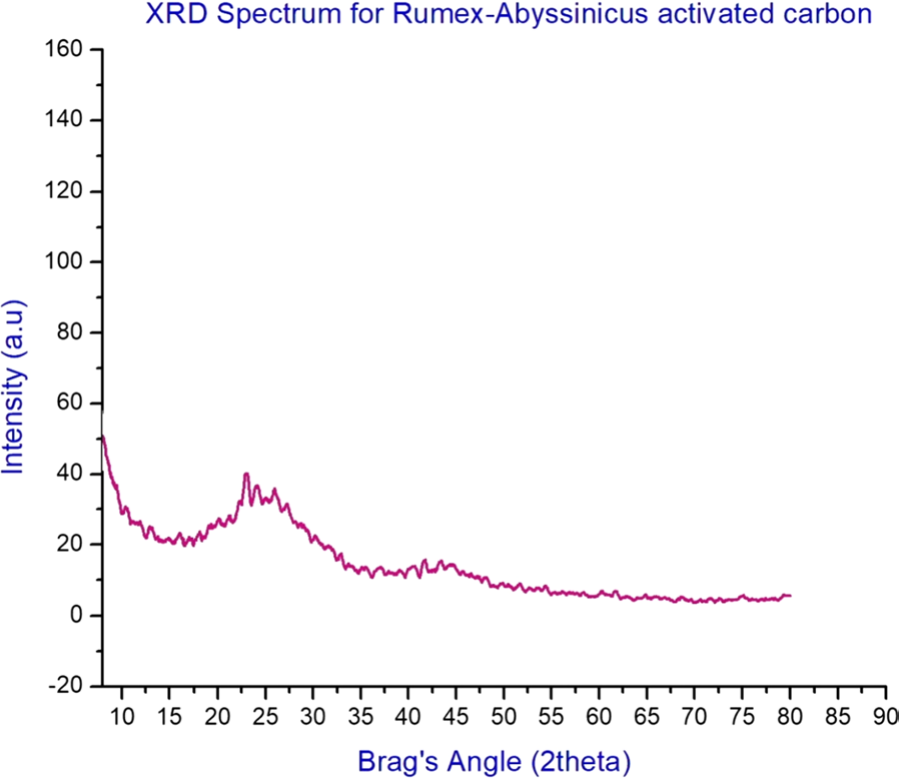
Crystalline structure analysis result for RAAC

The crystalline nature of RAAC was analyzed using X-ray diffraction and the result of the findings is indicated in Fig. 4. For this analysis, a scanning speed of 6 rev/min was guaranteed over an angle range of 10° to 80°. It was found that the prepared adsorbent has amorphous structure. The only significant peak observed during XRD analysis was at 2$\theta$ = 25. However, the general structure of the *Rumex abyssinicus* derived activated carbon can be deduced as an amorphous, resulting from the chemical and thermal activation process undertaken to enhance its adsorption capacity [6].

**Fig. 5:**
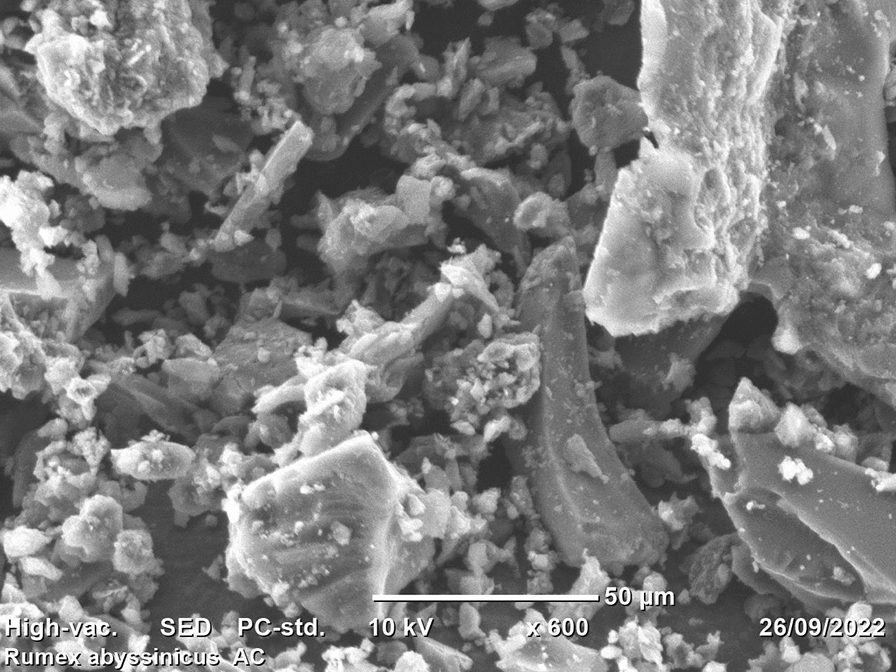
SEM morphology of RAAC

The morphological structure of activated carbon developed from the stem of *Rumex abyssinicus* was examined using (SEM) and its output is depicted in Fig. 5. During SEM analysis of examining the surface morphology, the surface of activated carbon adsorbent was magnified at a resolution of 600 times (600×), with pore size 50 μm at 15 kV. Basically, the intention of chemical activation as well as thermal activation is to create a porous carbonous material with a better carbon structure and high specific surface area. This porosity allows the attachment of the intended pollutant onto the morphological cracks observed. The SEM result of RAAC was found to have uneven distribution and irregular shape which creates a suitable condition for the adsorption of multipollutants varying in size and structure [6].

**Fig. 6:**
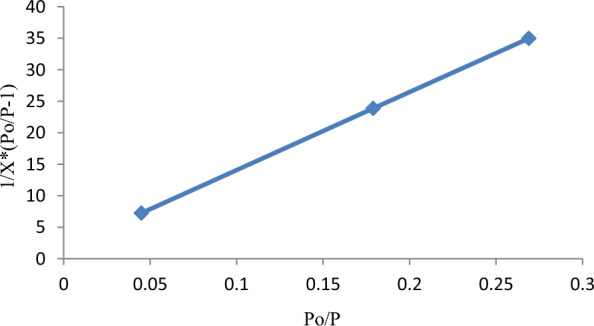
Nitrogen adsorption–desorption isotherm graph for BET surface determination

The BET—specific surface area of RAAC was determined using a principle of Nitrogen gas adsorption and desorption as indicated in Fig. 6. In this study, degassing temperature and time of 300 °C and 1 h were guaranteed. The currently synthesized *Rumex abyssinicus* derived activated carbon was found to have an extremely high specific surface area of 524 m^2^/g. The adsorbent’s high surface area is attributed to the nature of the precursor material, chemical activation, and thermal treatment processes conducted to enhance the adsorptive capacity of the material. Compared to many biomasses-based activated carbons, the RAAC was found superior in specific surface area. This higher BET surface area compared to other biomass based activated carbons is mainly attributed to the nature of the precursor material. Many researchers like [5, 67, 68] have also reported low BET surface areas of 426.8125 m^2^/g, 304.7250 m^2^/g, and 265 m^2^/g from Eucalyptus wood chips, arhar stalks, and parthenium hysterophorus, respectively.

### Batch adsorption of BBR dye

The batch adsorption study performed on the removal of BBR dye from an aqueous solution is presented in Table 6. The current study resulted in a maximum removal efficiency of 99.98% at optimum conditions of pH 2, contact time of 50 min, initial dye concentration of 100 mg/L, and adsorbent dosage of 0.15 g/100 mL. However, the minimum adsorption percentage of 51.32 was recorded at treatment conditions of pH 6, contact time 50 min, adsorbent dosage 0.05 g/100 mL and initial dye concentration 100 mg/L. On the other hand, the maximum and minimum adsorption capacity recorded throughout the study was 211.32 mg/g and 29.22 mg/g respectively. Moreover, the maximum adsorption capacity was recorded at pH 10, contact time of 80 min, initial dye concentration of 150 mg/L, and adsorbent dosage of 0.05 g/L. However, the minimum adsorption capacity was found at pH 6, dye concentration 50 mg/L, adsorbent dosage 0.15 g/100 mL, and contact time of 50 min. The maximum removal efficiency recorded in this study has reduced the amount of dye concentration from 100 to 0.02 mg/L, whereas at the maximum adsorption capacity, the pollutant concentration has reduced from 150 to 44.34 mg/L. This gives a clue that maximum adsorption capacity does not guarantee maximum removal efficiency and vice versa. Normally, adsorption capacity is highly dependent on the dosage of adsorbent used in which a small amount of adsorbent used for higher pollutant removal will result in higher adsorption capacity, whereas removal efficiency refers to the amount of pollutant reduction from wastewater or aquatic environment. Hence, the environmental issue is addressed by removal efficiency, whereas adsorption capacity is related to economics. The actual and predicted maximum removal efficiency is 99.98% and 99.57% respectively indicating the consistency of the experiment.

**Table 6:** Batch experiment results for adsorption of BBR dye from aqueous solution

Run no.	pH	Dye con. (mg/L)	Adsorbent dosage (g/100 mL)	Contact time (min)	Actual removal efficiency (%)	Predicted removal efficiency (%)	Adsorption capacity (mg/g)
1.	2	100	0.150	20	98.61	98.53	65.74
2.	2	100	0.100	20	93.38	92.16	93.38
3.	6	100	0.100	20	60.57	64.60	61.57
4.	2	150	0.100	20	97.15	99.53	145.73
5.	10	150	0.100	20	71.17	68.66	106.76
6.	6	50	0.050	20	80.17	79.42	80.17
7.	2	100	0.050	20	88.98	78.76	157.96
8.	6	150	0.150	20	75.38	75.16	75.38
9.	2	100	0.050	20	88.98	78.76	157.96
10.	6	150	0.100	20	65.82	65.64	98.73
11.	2	100	0.150	50	99.98	99.57	66.65
12.	10	50	0.150	50	96.72	99.43	32.24
13.	6	50	0.050	50	78.24	75.17	78.24
14.	2	100	0.050	50	86.45	83.33	172.9
15.	6	150	0.150	50	82.3	81.37	82.30
16.	6	100	0.050	50	51.32	53.85	102.64
17.	2	50	0.050	50	99.12	98.32	96.12
18.	10	50	0.100	50	97.85	97.69	48.93
19.	6	50	0.150	50	*77.68*	85.49	29.22
20.	10	100	0.050	50	58.86	61.28	117.72
21.	10	50	0.150	80	92.67	92.74	30.89
22.	2	100	0.050	80	93.52	90.80	187.04
23.	10	150	0.150	80	76.27	89.95	90.27
24.	10	50	0.100	80	92.2	92.77	47.1
25.	10	150	0.050	80	72.44	70.76	211.32
26.	10	50	0.050	80	88.55	85.77	86.55
27.	10	150	0.150	80	75.27	89.95	90.27
28.	6	150	0.150	80	88.81	90.49	88.81
29.	2	50	0.050	80	95.40	98.80	95.40
30.	6	150	0.1000	80	84.40	84.50	126.6

### Statistical analysis of variance (ANOVA)

#### Statistical model analysis and fit summary

The statistical analysis of the experimental data was conducted using RSM, and the analysis of variance (ANOVA) of the percentage removal was presented as in Table 7. The output of different models was compared, and focus was given to the non-aliased model, which maximized the adjusted R^2^ (0.9608) and the predicted R^2^ (0.9003) values and had additional terms that were significant. Furthermore, the two values agree, with a difference of less than 0.2. Therefore, the quadratic model was suggested.

**Table 7:** ANOVA for quadratic model

Source	Sum of squares	df	Mean square	F-value	p-value	Significance
Model	4717.57	14	336.97	51.78	< 0.0001	**
A-pH	1157.45	1	1157.45	177.85	< 0.0001	**
B-Dye concentration	278.35	1	278.35	42.77	< 0.0001	**
C-Adsorbent dosage	1041.46	1	1041.46	160.03	< 0.0001	**
D-Contact time	61.81	1	61.81	9.50	0.0076	**
AB	221.38	1	221.38	34.02	< 0.0001	*
AC	0.0583	1	0.0583	0.0090	0.9258	*
AD	19.65	1	19.65	3.02	0.1028	**
BC	83.68	1	83.68	12.86	0.0027	**
BD	319.76	1	319.76	49.13	< 0.0001	*
CD	17.14	1	17.14	2.63	0.1254	*
A^2^	1300.05	1	1300.05	199.76	< 0.0001	**
B^2^	543.86	1	543.86	83.57	< 0.0001	**
C^2^	45.03	1	45.03	6.92	0.0189	**
D^2^	9.02	1	9.02	1.39	0.2576	*
Residual	97.62	15	6.51			
Pure error	17.18	5	3.44			
Cor total	4815.19	29				
Lack of fit	97.62	13	7.51			*
C.V %	3.03					

**Significant terms and *insignificant terms

The quadratic regression model was found to be relevant for the response prediction, and an F-value of 57.78 indicates that the model is significant. P-values below 0.05 indicate that model terms such as A, B, C, D, AB, BC, BD, A^2^ and B^2^ are significant. However, *p*-values greater than 0.100 indicate that the model terms are not significant, in which case the model terms AC, AD, CD, C^2^ and D^2^ are insignificant. This implies a linear interaction between pH and dye concentration, adsorbent dosage and dye concentration, and dye concentration and contact time, and the quadratic interaction effect of pH, and dye concentration significantly affects the removal efficiency of dye.

The quadratic polynomial model was used to develop the mathematical relationship between the response and the independent process variables. The output of the fit summary of three different models is presented in Table 8. The selection of the best-fit model also focused on the adjusted R^2^ and the predicted R^2^ values being large, and additional terms being significant. The signal-to-noise ratio of 401.4, which is greater than 4, indicates that the model has an adequate signal and can be used to navigate the design space.

**Table 8:** Fit summary and Models’ lack of fit

Source	Sequential p-value	Lack of fit p-value	Adjusted R^2^	Predicted R^2^	Adeq. precision	
Linear	< 0.0075	0.0003	0.3223	0.2024		
2FI	0.6072	0.0002	0.2816	0.4247		
Quadratic	< 0.0001	0.2477	0.9608	0.9003	401.4	Suggested
Cubic	0.2477	–	0.9799			Aliased

Source	Sum of squares	Df	Mean square	F-value	P-value	
Linear	2874.01	20	143.70	41.83	0.0003	
2FI	2312.15	14	165.15	48.07	0.0002	
Quadratic	65.34	10	6.53	1.90	0.2477	Suggested
Cubic	0.000	0				Aliased
Pure error	17.18	5	3.44			

#### Empirical model by response surface estimation

The empirical model was developed using Box–Behnken Design approach of response surface for the maximum removal of reactive dye from wastewater, where a relationship was built among the four independent variables and the response variable using a second-order polynomial equation. Based on the model analysis, a quadratic model was fitted to the data model to predict the response variable. An empirical relationship between the response and the independent variables is shown by the following quadratic model Eq. 4.

4 $$ \begin{aligned} {\text{Removal}}\;{\text{Efficiency}}\;\left( \% \right) & = 65.58 - 10.93{\text{A}} - 5.11{\text{B}} + 8.21{\text{C}} + 2.44{\text{D}} - 6.33{\text{AB}} + 0.0921{\text{AC}} \\ & \quad - 1.82{\text{AD}} + 3.06{\text{BC}} + 6.99{\text{BD}} - 1.76{\text{CD}} + 18.45{\text{A}}^{2} + 13.15{\text{B}}^{2} - 3.51{\text{C}}^{2} + 1.45{\text{D}}^{2} , \\ \end{aligned} $$

where A is the solution pH, B is the dye concentration, C is the adsorbent dosage, The parameters A, B, C, D, AB, BC, BD, A^2^, and B^2^ were found to have significant effect on the response variable as the P-value for all is less than 0.05 (P < 0.05), with a relative significance of A^2^ > B^2^ > A > C > BD > AB > B > C^2^ > BC > D. However, P-values for AC, AD, CD, C^2^ and D^2^ are greater than 0.1000 indicating that the model terms are not significant. The model equation describes how the dye removal efficiency was affected by individual variables by their (linear and quadratic) terms or double interaction. Negative coefficients indicate that factors negatively affect the response variable. In this specific study, the double interaction effect of pH and dye concentration, dye concentration, and time is negatively affecting the response variable. Similarly, the quadratic effect of adsorbent dosage was antagonistically affecting the performances.

#### Adequacy checks for the developed model

**Fig. 7:**
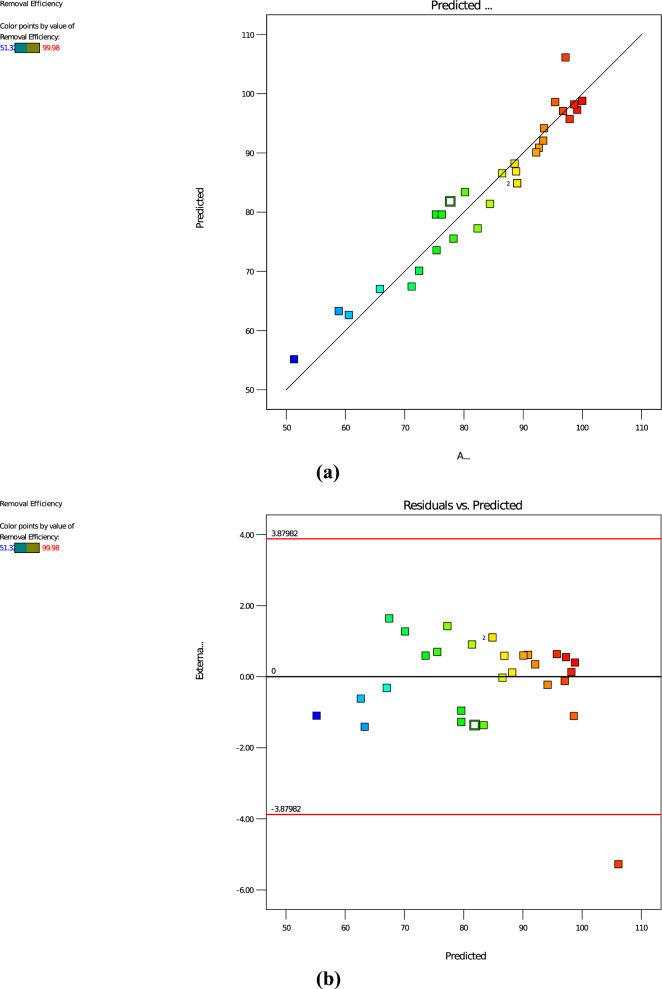
**a** Plot of actual versus predicted values and **b** residual versus predicted points for adsorption of reactive dye using RAAC

The goodness of fit of the model was checked using multiple correlation coefficients (R^2^). The quality of model fit for the adsorption process was evaluated using Fisher’s test (F-value), the probability value (p-value), the lack of fit, the coefficient of determination (R^2^), adjusted R^2^ (R^2^adj), and predicted R^2^ (R^2^pred). The F-value and P-value of the quadratic model were found to be 51.78 and 0.0001, respectively. This implies that the quadratic model is significant, and that the model can sufficiently predict the removal efficiency of reactive dye from aqueous environment. The quality of the fit was also examined by comparing the actual values against the predicted responses by the model of reactive dye removal shown in depicts that the predicted values are quite close to the actual experimental result, both spreading closer to the straight line, which indicates that the model fits the experimental data. Thus, it confirms that the regression model exhibits excellent stability for dye adsorption on activated carbon. Therefore, it can be concluded that the developed response surface model proved satisfactory for the prediction of the dye adsorption system. In addition to this, the adequacy of the model was also checked using residual analysis, as shown in Fig. 7a, b. The zero residual line in the plot was used to detect whether the points are scattered around the horizontal band or clustered in a curved pattern. In this specific study, the data points form a detectable pattern at almost the same distance from the zero residual line. Furthermore, the residual data points fluctuate more or less in a random fashion within the horizontal band, indicating that the model is desirable, and that there are no visible model defects.

### Interaction effect of factors on BBR dye removal efficiency

#### pH and initial dye concentration

**Fig. 8:**
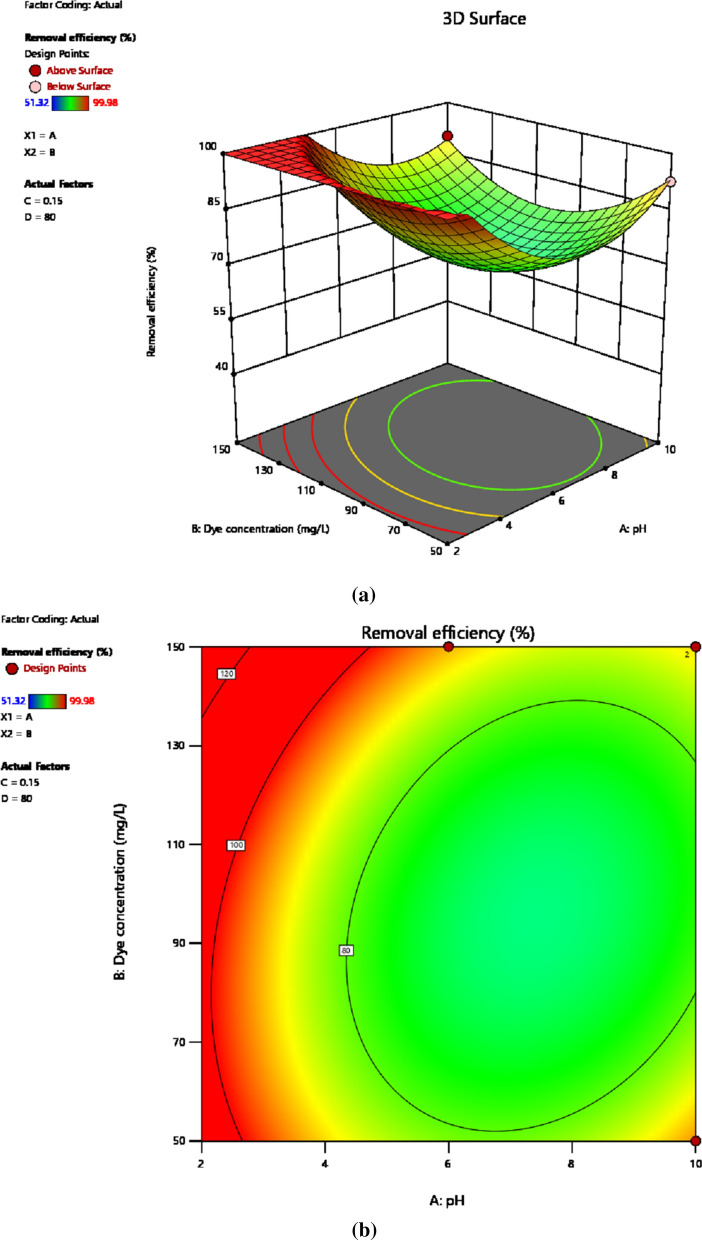
3D view (**a**) and Contour map (**b**) showing interaction effect of pH and initial dye concentration on dye removal efficiency

The interaction effect at varying levels of dye concentration and solution pH on the response variable is shown in the 3D model of Fig. 8a and contour map of Fig. 8b. Adsorption of intended pollutant onto the adsorbent material is affected by individual factor as well as the interaction among experimental variables. Hence, the interaction among experimental variables plays crucial role in the adsorption capacity as well as removal efficiency. Figure 8a, b depicts that both independent variables and their interaction strongly affect adsorption of the reactive dye and hence the removal efficiency. The interaction effect between the two variables was studied keeping adsorbent dosage and time of contact at 0.15 g/100 mL and 50 min, respectively. The 3D plot and contour map for the interaction effect shows that better removal efficiency was attained with the drop of solution pH and rise of the pollutant concentration where 100% removal efficiency was achieved at pH (2) and 100 mg/L of dye concentration. This increase of removal efficiency with dye concentration is due to driving force generated due to concentration gradient and increase in the probability of interaction of the pollutant molecules with the active sites on the adsorbent [69]. P-value of less than 0.001 reflects the presence of a relatively stronger interaction effect among the initial dye concentration and solution pH [70]. Beside this, an increase in protons due to the lowering of pH provides a suitable medium for the interaction of adsorbent active sites with the anionic textile dye molecules. This is because the chemistry of the dye molecules was affected by the solution pH and the activity of functional groups on the adsorbent surface [71]. Moreover, the competition of due molecules with hydrogen ions for the binding active sites was also observed.

#### Initial dye concentration and adsorbent dosage

**Fig. 9:**
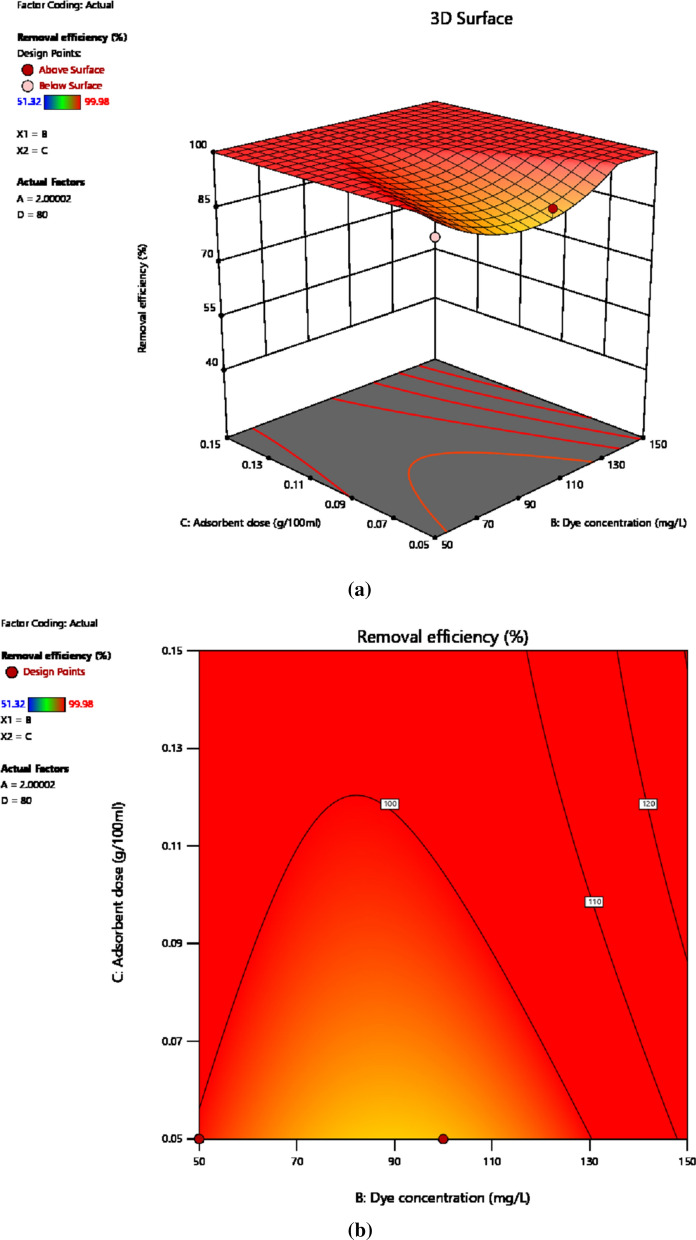
3D view (**a**) and Contour map (**b**) showing interaction effect of initial dye concentration and adsorbent dosage on dye removal efficiency

To evaluate the significance of the impact of linear interaction of initial dye concentration and adsorbent dosage the 3D view representation is as indicated by Fig. 9a, b, which were drawn holding pH and contact time at 2 and 80 min respectively. Herein, initial dye concentration and adsorbent dosage was varied from 50 to 150 mg/L and 0.05 to 0.15 mg/100 mL respectively. The maximum removal efficiency of dye resulted from the interaction of initial dye concentration and adsorbent concentration was found to be 100% which is detected mostly around higher values of dye concentration and adsorbent dosage. Whereas the minimum removal efficiency attained was below 90%, approximately around 60%. Normally, increasing the adsorbent dosage initially increase the adsorption efficiency however, further increase does not guarantee enhancement of adsorption capacity due to the availability of excessive active sites beyond the required optimum amount [6, 72, 73]. Generally, the removal efficiency of dye was positively affected by the interaction of adsorbent dosage and initial dye concentration.

#### Initial dye concentration and contact time

**Fig. 10:**
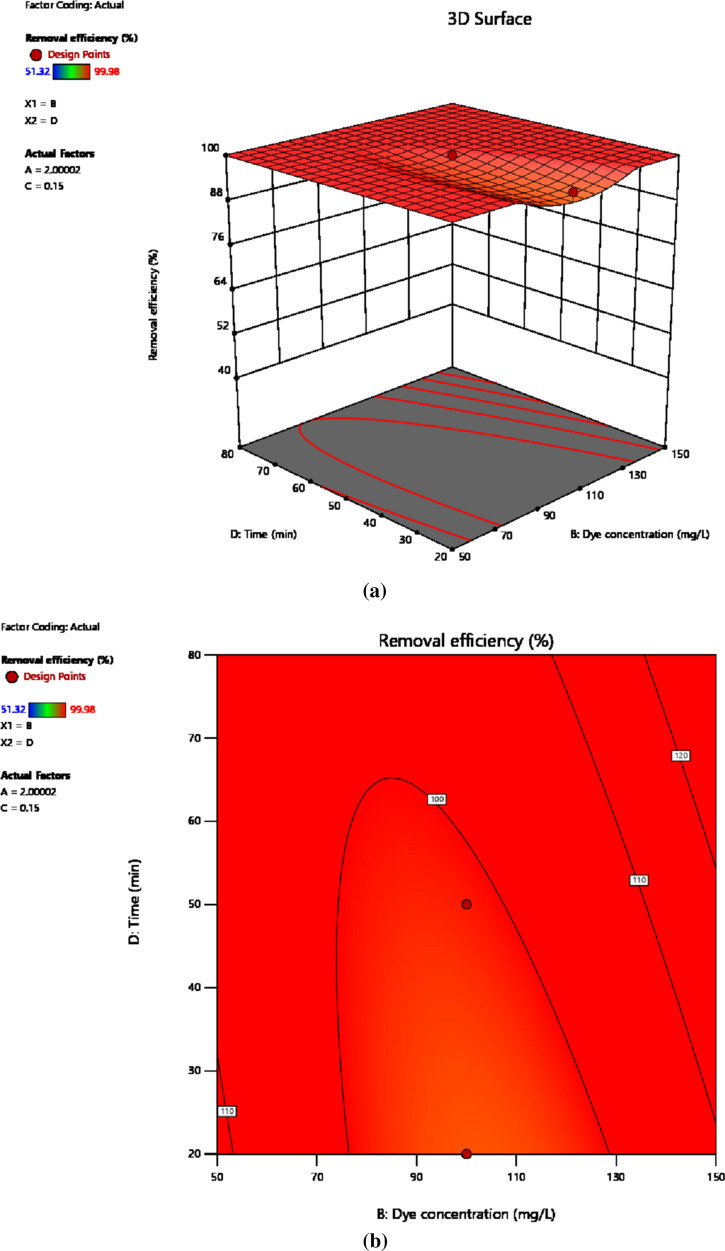
The response surface (**a**) and contour map (**b**) showing the interaction of dye concentration and contact time

The 3D plot and contour map in Fig. 10a, b shows the effect due to the interaction of dye concentration and contact time. The study of interaction among the two variables was done keeping pH 2 and adsorbent dosage (0.15 mg/100 mL) constant. The interaction effect of these parameters can be studied moving along the 100% contour line, which means that at the pollutant concentration of 125 mg/L the contact time required to achieve 100% removal efficiency is 80 min. On the other hand, when the dye concentration rises to 150 mg/L contact time needed reduced to 35 min. This has an indication that the concentration gradient, hence the probability of interaction of the dye molecules with the active sites on the adsorbent surface increases with concentration [69, 73]. This is because the more concentrated the dye the better the driving force and the frequency of contact with the adsorbent. However, lower pollutant concentration requires more time of contact in order to attain the 100% removal efficiency. Similarly, with the drop in the concentration of dye molecules below 125 mg/L, the time of contact required again increases due to a reduction in the probability of dye molecules interacting with the adsorbent active sites. In another situation, a drop in removal efficiency is observed due to a decrease in dye concentration at the constant contact time, which is due to a reduction in the frequency of contact with the fixed amount of adsorbent. In general, the interaction of dye concentration and time of contact affects the response variable negatively.

### Adsorption isotherm

**Fig. 11:**
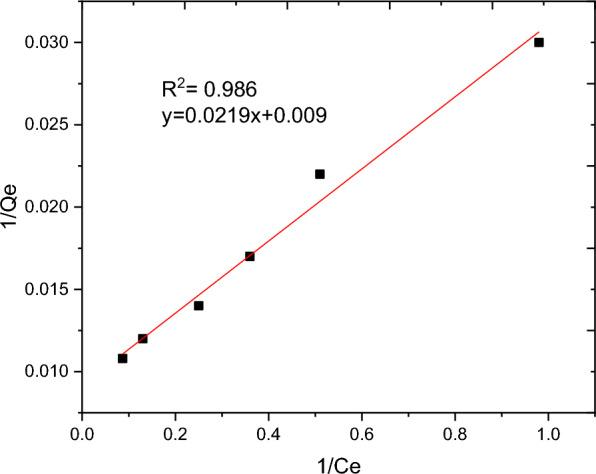
Graphical representation of Langmuir isotherm (linear) for adsorption of BBR dye

**Fig. 12:**
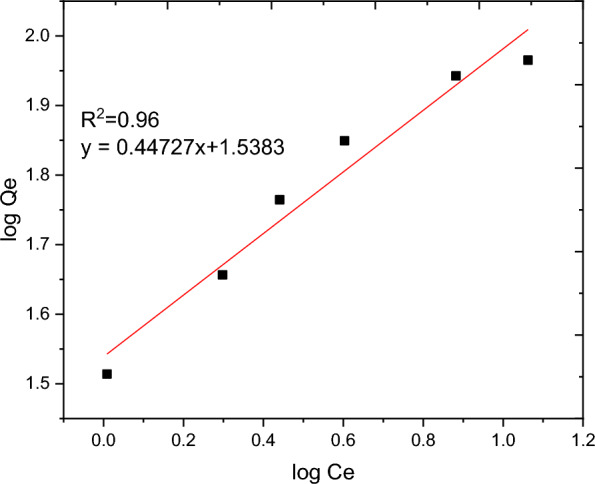
Freundlich isotherm (linear) for BBR dye adsorption onto RAAC

**Fig. 13:**
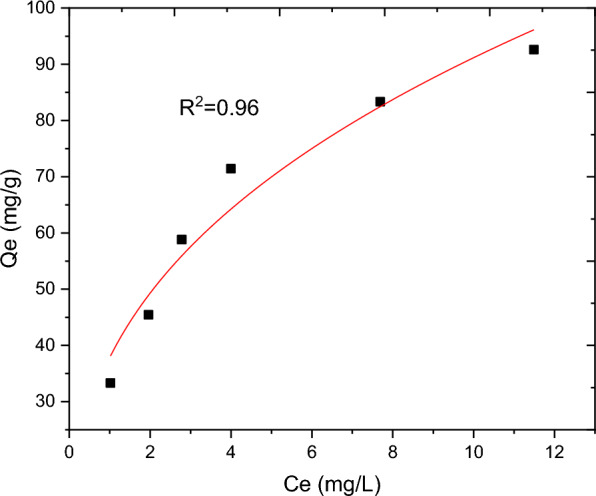
Freundlich isotherm (non-linear) for BBR dye adsorption onto RAAC

**Fig. 14:**
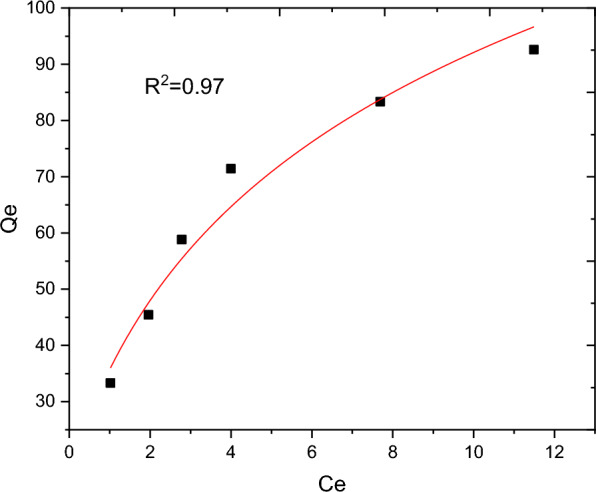
Non-linear form of Temkin isotherm model for adsorption of BBR dye

**Fig. 15:**
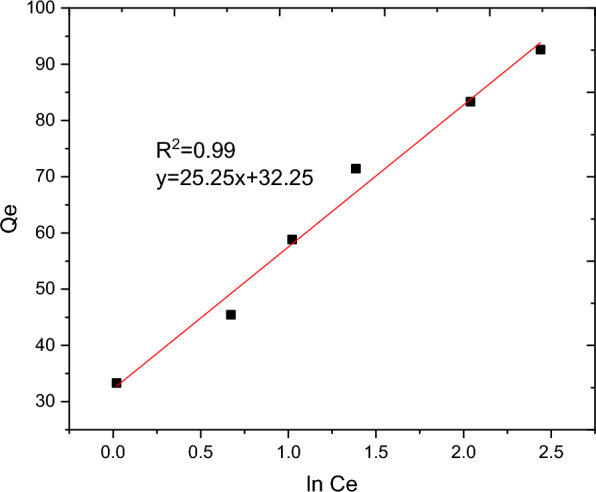
Linearized form of Temkin isotherm model for BBR dye adsorption

**Fig. 16:**
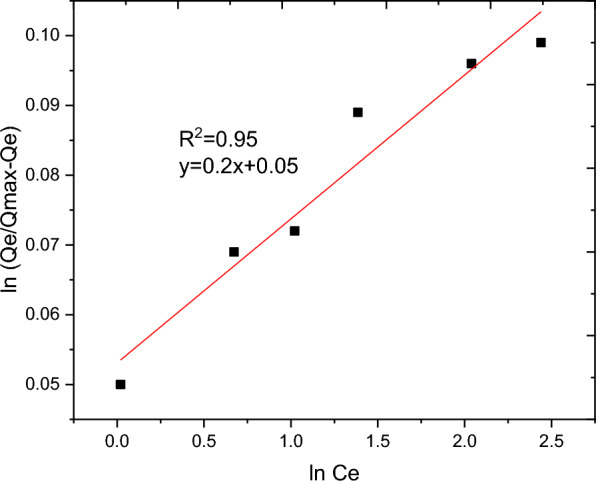
Linear form of Sips isotherm model for removal of BBR dye from aqueous solution

The nature of interaction between the adsorbent and adsorbate at equilibrium was investigated using the Langmuir, Freundlich, Temkin and Sips adsorption isotherm models. These models’ constants as well as the coefficient of determinant R^2^ are presented in Table 9. Moreover, the linear plots resulting from 1/qt vs 1/Ce for Langmuir and log Qe vs log Ce for Freundlich isotherms are shown in Figs. 11 and 12, respectively. It can be observed from Fig. 11 that the R^2^ value for linearized Langmuir type of isotherm model was found to be 0.99. Additionally, Q_max_ and K_L_ value obtained from the linear equation resulted from plot was determined to be 108.69 mg/g and 45.662 L/g, respectively. The R_L_ value recorded from this study was found to be 0.02, indicating a favorable condition. On the other hand, the R^2^ and 1/n determined from linear Freundlich isotherm was found to be 0.96 and 0.44, indicating a favorable adsorption. The non-linear forms of adsorption isotherm models; Freundlich (Fig. 13) and Temkin (Fig. 14) are almost resulted in similar degree of fits with their linear forms (Figs. 12 and 15 respectively). Linear forms of Langmuir (Fig. 11) and Sips (Fig. 16) were found to fit the data despite their non-linear forms lack of convergence. In comparison, the Langmuir isotherm model (linear form) was found to be more explanatory, suggesting the nature of the interaction to be monolayer and homogenous. This implies the surface of the adsorbent can be saturated with single attachment of the dye.

**Table 9:** Linear and non-linear constant s for various adsorption isotherm studies

Isotherm model	Linear constants	Non- linear constants
Langmuir	R^2^ = 0.99 Qmax = 108.69 mg/g KL = 45.66 L/mg R_L_ = 0.02 Residual sum of squares = 3.65 × 10^–6^	Does not converge at all
Freundlich	R^2^ = 0.96 Kf = 34.59((mg/g)(L/mg))^0.25^ 1/n = 0.44 n = 2.27 Residual sum of squares = 0.00594	R^2^ = 0.96 Kf = 37.84((mg/g)(L/mg))^0.25^ 1/n = 0.38 n = 2.63 Reduced chi-square = 27.10
Temkin	R^2^ = 0.99 bT = 98.17 kT = 3.585 Residual sum of squares = 34.6	R^2^ = 0.97 bT = 37.69 kT = 1.59 Reduced chi-square = 21.41
Sips	R^2^ = 0.95 1/ns = 0.2 ns = 5 ks = 29.68 Residual sum of squares = 0.00095	Does not converge at all

### Adsorption kinetics

**Fig. 17:**
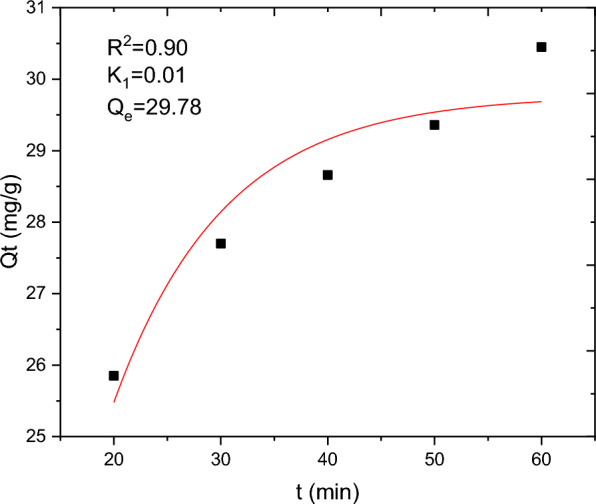
PFO kinetics (non-linear) plot for BBR dye adsorption from aqueous solution

**Fig. 18:**
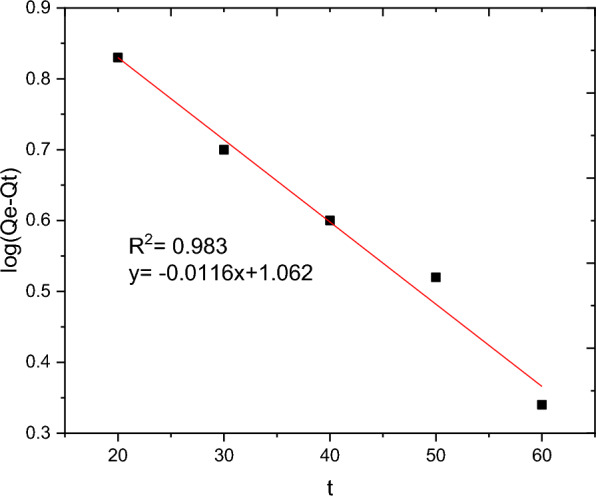
PFO kinetics (linear) for BBR dye uptake form aqueous solution

**Fig. 19:**
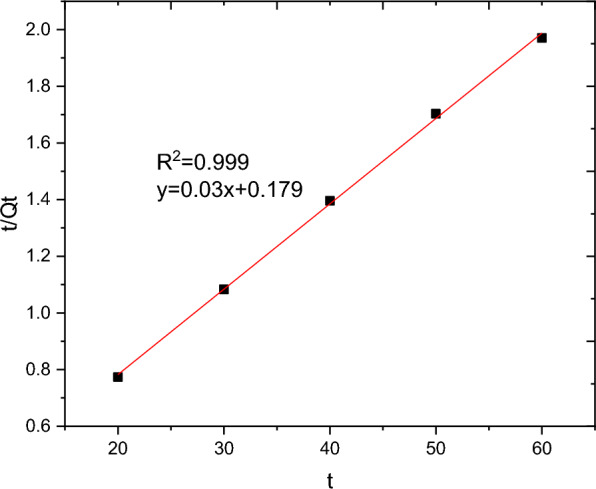
PSO kinetics (linear plot) for BBR dye adsorption from aqueous solution

**Fig. 20:**
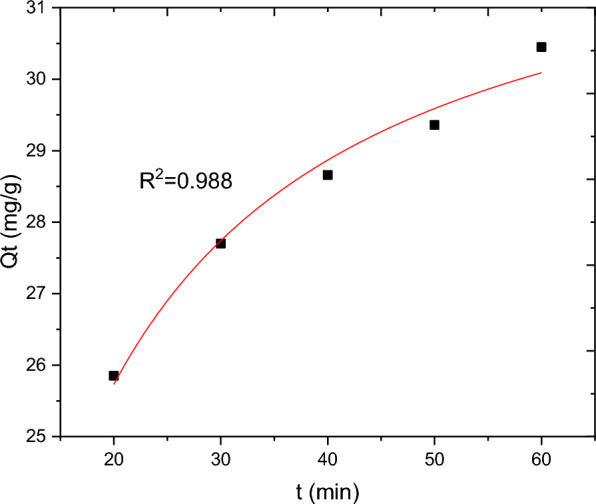
PSO kinetics (non-linear plot) for BBR dyes adsorption from aqueous solution

**Fig. 21:**
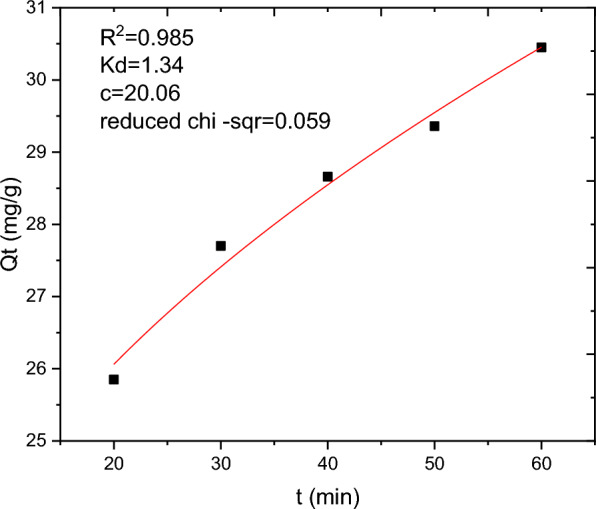
IPD non-linear plot for BBR dye adsorption from aqueous solution

**Fig. 22:**
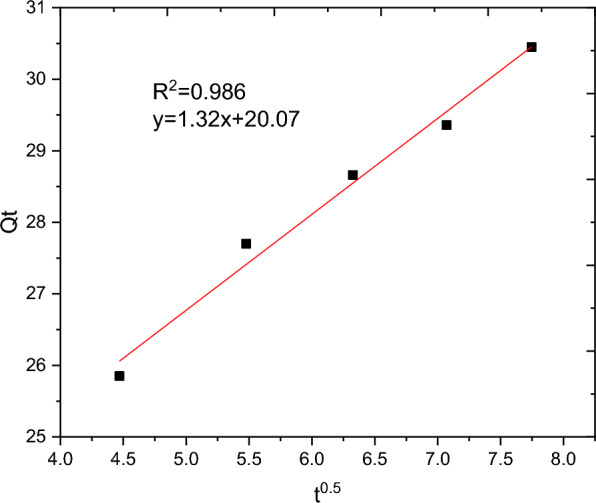
IPD kinetics plot (linear form) for BBR dye adsorption from aqueous solution

**Fig. 23:**
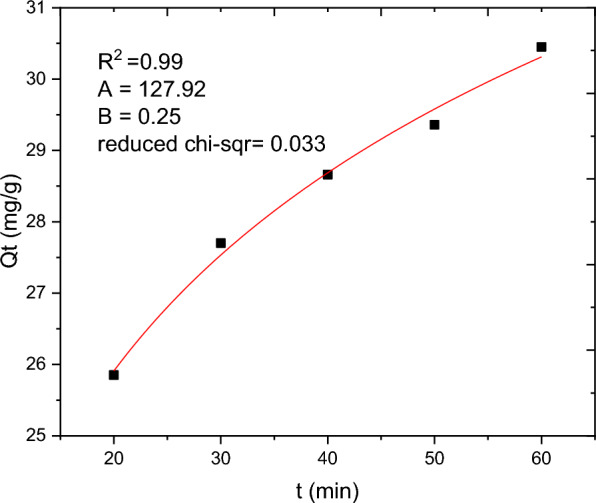
Elovich kinetics model (non-linear) for BBR dye removal from aqueous solution

**Fig. 24:**
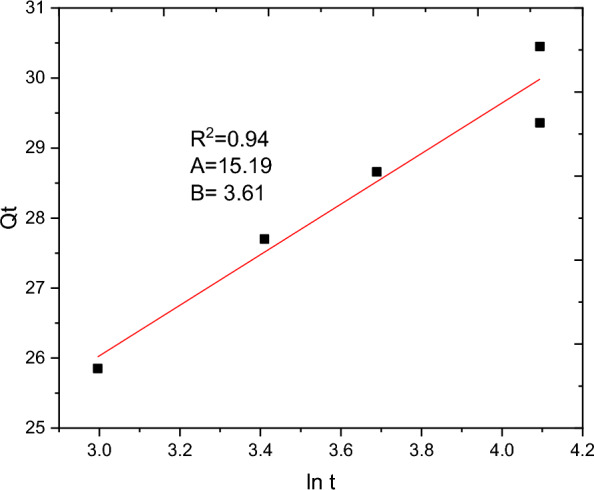
Elovich kinetics (linear form) plot for BBR adsorption from aqueous solution

The batch adsorption experimental data are fitted with PFO, PSO, IPD and Elovich kinetics models and the results are presented in Table 10. The selection of the best data fit was evaluated using determinant coefficient, as well as the error analysis was carried using residual sum of squares (for linear plot) and reduced chi square (for non-linear plot). The non-linear and linear plots of PFO kinetics models are presented in Figs. 17 and 18 respectively. It seems the non-linear equation of PFO with R^2^ = 0.98 fits better when compared to the linear one (R^2^ = 0.90). On the other hand, Figs. 19 and 20 depict the non-linear and linear forms of PSO models respectively. Kinetics constants (R^2^, K_2_, and Qe, calculated) for linear and non -linear plots of PSO are almost equal. Additionally, the linear and non-linear plots of IPD model are depicted in Figs. 21 and 22 respectively. The application Elovich kinetic model for analysis of BBR dye adsorption onto activated carbon of *Rumex abyssinicus* was carried both in non-linear and linear forms and depicted in Figs. 23 and 24 respectively. In comparison, PSO (linear) with a maximum R^2^ of 0.99, insignificant difference between Qe, experimental (32.66 mg/g) and Qe, calculated (33.22 mg/g) and very small discrepancy between the data and estimated value was found descriptive. Hence, the adsorption process is chemisorption with strong bond and slow attachment process between adsorbate and adsorbent and saturated sites exist and the reaction rate is affected by the concentration of BBR dye and RAAC.

**Table 10:** Kinetics models for adsorption of BBR dye onto RAAC

Kinetics models	Linear constants	Non-linear constants
PFO	R^2^ = 0.98 K_1_ = 0.0267 min^−1^ Qe, calculated = 11.53 mg/g Residual sum of squares = 0.00232	R^2^ = 0.90 K_1_ = 0.01 min^−1^ Qe, calculated = 29.78 mg/g Reduced chi-square = 0.025
PSO	R^2^ = 0.99 K_2_ = 0.005 g/mg min Qe, calculated = 33.22 mg/g Residual sum of squares = 7.6 × 10^–4^	R^2^ = 0.99 K_2_ = 0.006 g/mg min Qe, calculated = 33.18 mg/g Reduced chi-square = 0.0004
IPD	R^2^ = 0.98 K_d_ = 1.34 mg/g.min^0.5^ C = 20.07 mg/g Residual sum of squares = 0.176	R^2^ = 0.98 K_d_ = 1.34 mg/g.min^0.5^ C = 20.06 mg/g Reduced chi-square = 0.059
Elovich	R^2^ = 0.94 α = 0.25 β = 129.19 Residual sum of squares = 0.1006	R^2^ = 0.99 α = 0.25 β = 127.92 Reduced chi-square = 0.033

### Regeneration and reusability study

**Fig. 25:**
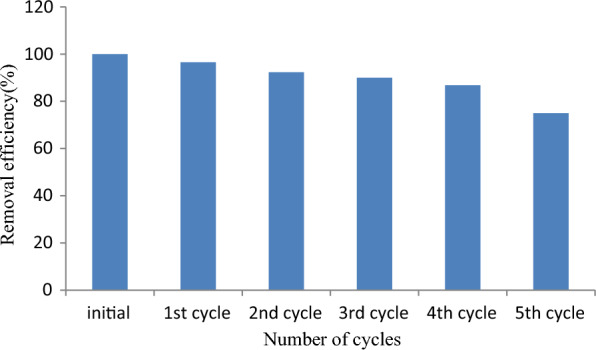
Adsorbent desorption and its BBR dye removal potential

A regeneration and reusability study of the spent adsorbent is of immense importance since it provides information whether lab scale production is applicable or not for mass production at an industrial scale. Hence, the regeneration of RAAC was conducted through a chemical method and tested for practical reusability for five cycles. Precisely, 0.1 g of pollutant loaded material was shaken at 80 rpm for 2 h in NaOH (0.1 M and 100 mL). Then, after the pollutant was significantly desorbed, the filtration of the solution was carried out using Whatman filter paper. Therefore, the desorbed material was dried in an oven and used for five cycles for examining its reusability potential. The result of the reusability study is depicted in Fig. 25 and the removal capability of the reused RAAC was found encouraging with BBR dye removal efficiency ranging from 96.5 to 75.01%. Hence, the results suggest that RAAC was found to possess high reusability potential which in turn could give it great potential to be used at an industrial level.

### Cost analysis

The cost for laboratory scale produced *Rumex abyssinicus* derived activated carbon includes costs for raw material preparation, sample collection, raw material drying, chemical activation, thermal activation, neutralization and drying of activated carbon. The total production cost was estimated to be 85.41 USD/kg. This production cost is approximately three times cheaper than commercial activated carbon with a high specific surface area, which is 259.5 USD/kg [74]. Hence, RAAC can serve as alternative toxic dye removal with low cost. Finally, Table 11 is comprehensive table inclusive of all the lab scale cost involved during the fabrication of current activated carbon.

**Table 11:** Simple cost analysis for laboratory scale produced RAAC

Activity	Cost
Sample collection	80 ETB, half-day work for one labour worker
Raw material drying	0, used sun-dried *Rumex abyssinicus*
Size reduction	2.29 ETB, KWh = 1.223 ETB, the grinding machine utilizes 0.75 KWh per hour, takes 0.5 to 3 min to grind 50 g of the *Rumex abyssinicus*, 780 g of *Rumex abyssinicus* end up with 314 g of activated carbon, which means 2.5 kg of the raw material was required to produce 1 kg of activated carbon, 2.5 h was used to finish grinding the RM, that means (0.75 KWh * 2.5 h)/h = 1.875 KWh * 1.223ETB = 2.29ETB
Chemical activation	Cost of 88% phosphoric acid in Ethiopia per liter is 800ETB, diluted to 50% phosphoric acid solution for application due to high moisture uptake of the raw material, the specific gravity of 50% phosphoric acid = 1.334 kg/L and 600 ETB used per kg. Hence, to impregnate 2.5 kg of raw *Rumex abyssinicus* sample needed 2.5 kg * 3 * 600 ETB = 4500ETB
Thermal activation	Furnace energy consumption 26 KWh per day, for 2 h of activation 2.167 KWh * 1.223ETB/KWh = 2.65 ETB
Neutralization	Distilled water was used for neutralization, approximately 10 ETB
Drying of activated carbon	0.75 KWh/h * 8 * 1.23 ETB = 7.38 ETB

### Comparative study

Compared to similar classes of activated carbons produced from biomass materials, RAAC applied for the removal of BBR dye found to be superior. The improvement may be described in terms of small amount adsorbent consumption for substantial pollutant detoxification, which is related to adsorption capacity, performing well at low contact time, which is used to minimize the time spent, and more importantly the maximum removal efficiency recorded from an environmental perspective makes the current study promising. Table 12 presents performance of different biomass based activated carbons in adsorption of brilliant blue reactive dye.

**Table 12:** Comparative study of RAAC with other adsorbents applied for removal of BBR dye

S no.	Activated carbon precursor	Treatment conditions	Removal efficiency/adsorption capacity	References
1.	Coconut shell	Adsorbent dosage 5 g/L, 360 min contact time, initial dye concentration 10 mg/L and pH 2	98.77%	[75]
2.	Macroporous polystyrene resin	Solution pH 2.0, 150 min contact time at 25 °C, and adsorbent dosage 1.0 g/L	208.33 mg/g	[76]
3.	Snail shell powder	Contact time 10 min, dye concentration 30 mg/L, 0.01 g/100 mL	99.09%	[77]
4.	Date pits	1.5 g/L adsorbent dosage, 40 mg/L dye concentration, pH of 2 and contact time of 50 min	90.4%	[78]
5.	Gelidium corneum biomass	100 mg/L RBB, pH 5, 4 g/L algal biomass and 180 min of contact time	89.18%	[19]
6.	Pomegranate fruit peel	Adsorbent dose 0.2 g/200 mL, dye concentration 25 mg/L, contact time 24 h	81.35%	[79]
7.	Sewage sludge biochar	Adsorbent dose of 100 g/L and dye concentration (100 mg/L) after 60 min	87.03 mg/g	[18]
8.	Thuja orientalis leaves:	pH 6 and contact time of 300 min, adsorbent dosage 2 g/L and dye concentration 4.165 g/L	81%	[16]
9.	*Rumex abyssinicus* derived activated carbon	pH of 2, contact time 50 min, dye concentration 100 mg/L and adsorbent dosage of 0.15 g/L	99.98%	Current study

## Conclusion

*Rumex abyssinicus*-based activated carbon was developed and used for the adsorption of BBR dye in aqueous solutions, where the adsorbent material was prepared through chemical activation followed by thermal activation. Proximate analysis, pHpzc, SEM, FTIR, XRD, and BET techniques were used to characterize the developed biomass-derived activated carbon, and the analysis result showed that the adsorbent is composed of 3.4% moisture content, 9.5% ash content, 16.2% volatile matter, and a fixed carbon percentage of 70.9, indicating the quality of the prepared precursor material. On the other hand, the pHpzc was determined to be 6.90, which describes that the surface of the adsorbent is positively and negatively charged below and above pH 6.9, respectively. The SEM analysis resulted in irregular porous morphology and the presence of multiple functional groups were analysed using FTIR. Normally, these multiple functional groups along with the porous, non-uniform shapes of the adsorbent enhance the adsorption of multipollutant of varying in size. The high BET specific surface area of 524 m^2^/g as well as the amorphous surface structure of the adsorbent has made the synthesized activated carbon a potential candidate for the removal of toxic dyes like BBR. Response surface methodology coupled with the Box Behnken approach was used to design the experiment, where four experimental variables with three levels: pH (2, 6, and 10), initial dye concentration (50, 100, and 150 mg/L), adsorbent dosage (0.05, 0.1, and 0.15 g/100 mL), and contact time (20, 50, and 80 min) were used to generate 30 experimental runs. Batch adsorption experiments resulted in maximum and minimum removal efficiency of 99.98 and 51.32%, respectively, where the maximum removal efficiency of 99.98% was obtained at optimum batch adsorption conditions of pH 2, contact time of 50 min, initial dye concentration of 100 mg/L, and adsorbent dosage of 0.15 g/100 mL. Among the regression models, a quadratic regression model with R^2^ of 0.9797 was found to better describe the data. Moreover, the maximum predicted removal efficiency of 99.57% was obtained using the quadratic model, which is very close to the experimentally determined value of 99.98%. On the other hand, the interaction effects involved between pH and initial dye concentration, initial dye concentration and adsorbent dosage, and initial dye concentration and contact time were found to be significant. Among adsorption isotherms, the Langmuir isotherm model with R^2^ of 0.99 was found to be explanatory, indicating the interaction of adsorbent and adsorbate monolayer and homogenous. In addition to this, pseudo-second order kinetics with an insignificant difference between Q experimental and Q calculated and a R^2^ of 0.99 were found to fit the data well, suggesting the nature of the adsorption to be chemosorption, in which both adsorbate and adsorbent concentrations are potential rate-determining factors. Finally, it can be inferred that *Rumex abyssinicus*-derived activated carbon appears to be a potential candidate for the detoxification of harmful dye-containing effluent. However, further investigation, such as column and thermodynamics studies, is recommended befor**e** applying at an industrial level for wastewater treatment.

## Data Availability

All data are fully available without restriction from the corresponding author at any time through email.

## References

[1] Uddin J, Jeong Y (2021). Urban river pollution in Bangladesh during last 40 years: potential public health and ecological risk, present policy, and future prospects toward smart water management. Heliyon.

[2] Zamora-ledezma C (2021). Heavy metal water pollution: a fresh look about hazards, novel and conventional remediation methods. Environ Technol Innov.

[3] Shah MI, Javed MF, Abunama T (2021). Proposed formulation of surface water quality and modelling using gene expression, machine learning, and regression techniques. Environ Sci Pollut Res.

[4] Moges A, Nkambule TTI, Fito J (2022). The application of GO-Fe_3_O_4_ nanocomposite for chromium adsorption from tannery industry wastewater. J Environ Manag.

[5] Bedada D, Angassa K, Tiruneh A, Kloos H, Fito J (2020). Chromium removal from tannery wastewater through activated carbon produced from *Parthenium hysterophorus* weed. Energy Ecol Environ.

[6] Fito J, Abewaa M, Nkambule T (2023). Magnetite-impregnated biochar of *Parthenium hysterophorus* for adsorption of Cr(VI) from tannery industrial wastewater. Appl Water Sci.

[7] Geng M (2021). Evaluation and variation trends analysis of water quality in response to water regime changes in a typical river-connected lake (Dongting Lake), China. Environ Pollut.

[8] Lin L, Yang H, Xu X (2022). Effects of water pollution on human health and disease heterogeneity: a review. Front Environ Sci.

[9] Liu L (2021). The use of GIS-based genetic algorithm in water pollution control planning. Desalin Water Treat.

[10] Lin L, Yang H, Xu X (2022). Effects of water pollution on human health and disease heterogeneity: a review. Front Environ Sci.

[11] Liu HY, Jay M, Chen X (2021). The role of nature-based solutions for improving environmental quality, health and well-being. Sustainability.

[12] Samarghandi MR, Dargahi A, Shabanloo A, Nasab HZ, Vaziri Y, Ansari A (2020). Electrochemical degradation of methylene blue dye using a graphite doped PbO_2_ anode: optimization of operational parameters, degradation pathway and improving the biodegradability of textile wastewater. Arab J Chem.

[13] Achparaki M (2012). We are IntechOpen, the world’s leading publisher of Open Access books built by scientists, for scientists TOP 1 %.

[14] El Harfi S, El Harfi A (2017). Classifications, properties and applications of textile dyes: a review. Appl J Environ Eng Sci.

[15] Yadav A, Dindorkar SS (2022). Adsorption behaviour of hexagonal boron nitride nanosheets towards cationic, anionic and neutral dyes: insights from first principle studies. Colloids Surf A Physicochem Eng Asp.

[16] Chandra M (2020). Adsorptive removal of Remazol Brilliant Blue R dye from its aqueous solution by activated charcoal of *Thuja orientalis* leaves: an eco-friendly approach. SN Appl Sci.

[17] Mohamed WAA (2022). Degradation of local brilliant blue R dye in presence of polyvinylidene fluoride/MWCNs/TiO_2_ as photocatalysts and plasma discharge. J Environ Chem Eng.

[18] Raj A, Yadav A, Prakash A, Kumar A (2021). Kinetic and thermodynamic investigations of sewage sludge biochar in removal of Remazol Brilliant Blue R dye from aqueous solution and evaluation of residual dyes cytotoxicity. Environ Technol Innov.

[19] El-Ahmady Ali El-Naggar N, Hamouda RA, El-Khateeb AY, Rabei NH (2021). Biosorption of cationic H and Remazol Brilliant Blue anionic dye from binary solution using *Gelidium corneum* biomass. Sci Rep.

[20] Caicedo C (2019). Legionella occurrence in municipal and industrial wastewater treatment plants and risks of reclaimed wastewater reuse: review. Water Res.

[21] Sreedharan V, Krithishna KV, Nidheesh PV (2017). Removal of chromium and iron from real textile wastewater by sorption on soils. J Hazard Toxic Radioact Waste.

[22] Kaci MM (2022). Insights into the optical and electrochemical features of CuAl_2_O_4_ nanoparticles and it use for methyl violet oxidation under sunlight exposure. Opt Mater.

[23] Liu H, Zhang J, Lu M, Liang L, Zhang H, Wei J (2020). Biosynthesis based membrane filtration coupled with iron nanoparticles reduction process in removal of dyes. Chem Eng J.

[24] Gan C, Tuo B, Wang J, Tang Y, Nie G, Deng Z (2023). Photocatalytic degradation of reactive brilliant blue KN-R by Ti-doped Bi_2_O_3_. Environ Sci Pollut Res.

[25] Begum S (2020). Remarkable photocatalytic degradation of Remazol Brilliant Blue R dye using bio-photocatalyst ‘nano-hydroxyapatite’. Mater Res Express.

[26] Mohamed WAA (2022). Degradation of local Brilliant Blue R dye in presence of polyvinylidene fluoride/MWCNTs/TiO_2_ as photocatalysts and plasma discharge. J Environ Chem Eng.

[27] Fito J, Said H, Feleke S, Worku A (2019). Fluoride removal from aqueous solution onto activated carbon of *Catha edulis* through the adsorption treatment technology. Environ Syst Res.

[28] Rizzo L (2019). Consolidated vs new advanced treatment methods for the removal of contaminants of emerging concern from urban wastewater. Sci Total Environ.

[29] Inyang MI (2016). A review of biochar as a low-cost adsorbent for aqueous heavy metal removal. Crit Rev Environ Sci Technol.

[30] You Y, Shi Z, Li Y, Zhao Z, He B, Cheng X (2021). Magnetic cobalt ferrite biochar composite as peroxymonosulfate activator for removal of lomefloxacin hydrochloride. Sep Purif Technol.

[31] Akkari I, Graba Z, Bezzi N, Vithanage M, Kaci MM (2022). New insights into the effective removal of Basic Red 46 onto activated carbon produced from pomegranate peels. Biomass Convers Biorefin.

[32] Akkari I (2023). Effective removal of cationic dye on activated carbon made from cactus fruit peels: a combined experimental and theoretical study. Environ Sci Pollut Res.

[33] Jawad AH, Abdulhameed AS, Kashi E, Yaseen ZM, ALOthman ZA, Khan MR (2022). Cross-linked chitosan-glyoxal/kaolin clay composite: parametric optimization for color removal and COD reduction of Remazol Brilliant Blue R dye. J Polym Environ.

[34] Zhang H (2022). Efficient removal of Remazol Brilliant Blue R from water by a cellulose-based activated carbon. Int J Biol Macromol.

[35] Fito J, Abrham S, Angassa K (2020). Adsorption of methylene blue from textile industrial wastewater onto activated carbon of *Parthenium hysterophorus*. Int J Environ Res.

[36] Fito J, Tefera N, Van Hulle SWH (2017). Adsorption of distillery spent wash on activated bagasse fly ash: kinetics and thermodynamics. J Environ Chem Eng.

[37] Kassahun E (2022). The application of the activated carbon from *Cordia africana* leaves for adsorption of chromium (III) from an aqueous solution. J Chem.

[38] Mulisa E, Asres K, Engidawork E (2015). Evaluation of wound healing and anti-inflammatory activity of the rhizomes of *Rumex abyssinicus* J. (Polygonaceae) in mice. BMC Complement Altern Med.

[39] Mekonnen T, Urga K, Engidawork E (2010). Evaluation of the diuretic and analgesic activities of the rhizomes of *Rumex abyssinicus* Jacq in mice. J Ethnopharmacol.

[40] Getie M (2003). Evaluation of the anti-microbial and anti-inflammatory activities of the medicinal plants *Dodonaea viscosa*, *Rumex nervosus* and *Rumex abyssinicus*. Fitoterapia.

[41] Kengne IC (2021). Antibacterial, antifungal and antioxidant activities of whole plant chemical constituents of *Rumex abyssinicus*. BMC Complement Med Ther.

[42] Mohammed S, Gonfa G (2022). Adsorption of Cr(V) from aqueous solution using eggshell-based cobalt oxide-zinc oxide nano-composite. Environ Chall.

[43] Neme I, Gonfa G, Masi C (2022). Results in materials preparation and characterization of activated carbon from castor seed hull by chemical activation with H_3_PO_4_. Results Mater.

[44] Roba B, Yilma M, Abay Y, Mekonnen A (2023). A novel approach for the defluorination of groundwater using trivalent-metal hydroxide/bone-char composite adsorbent. Urban Clim.

[45] Akkari I (2022). Biosorption of Basic Red 46 using raw cactus fruit peels: equilibrium, kinetic and thermodynamic studies. Biomass Convers Biorefin.

[46] Akkari I, Spessato L, Graba Z, Bezzi N, Kaci MM (2023). A sustainably produced hydrochar from pomegranate peels for the purification of textile contaminants in an aqueous medium. Sustain Chem Pharm.

[47] Shahbazi D, Mousavi SA, Nayeri D (2020). Low-cost activated carbon: characterization, decolorization, modeling, optimization and kinetics. Int J Environ Sci Technol.

[48] Graba Z, Akkari I, Bezzi N, Kaci MM (2022). Valorization of olive–pomace as a green sorbent to remove Basic Red 46 (BR46) dye from aqueous solution. Biomass Convers Biorefin.

[49] Alireza S, Zangeneh H, Almasi A, Nayeri D (2020). Decolourization of aqueous Methylene Blue solutions by corn stalk: modeling and optimization. Desalination Water Treat.

[50] Mousavi SA, Shahbazi D, Mahmoudi A, Mohammadi P, Massahi T (2021). Statistical modeling and kinetic studies on the adsorption of Reactive Red 2 by a low-cost adsorbent: grape waste-based activated carbon using sulfuric acid activator-assisted thermal activation. Adsorpt Sci Technol.

[51] Ambika S (2022). Modified biochar as a green adsorbent for removal of hexavalent chromium from various environmental matrices: mechanisms, methods, and prospects. Chem Eng J.

[52] Liao J, Ding L, Zhang Y, Zhu W (2022). Efficient removal of uranium from wastewater using pig manure biochar: understanding adsorption and binding mechanisms. J Hazard Mater.

[53] Chen J, Yang R, Zhang Z, Wu D (2022). Removal of fluoride from water using aluminum hydroxide-loaded zeolite synthesized from coal fly ash. J Hazard Mater.

[54] Adane T, Adugna AT, Alemayehu E (2022). Bentonite blended with bagasse ash as an adsorbent for reactive red 198 dyes. Water Pract Technol.

[55] Adane T, Hailegiorgis SM, Alemayehu E (2022). Acid-activated bentonite blended with sugarcane bagasse ash as low-cost adsorbents for removal of reactive red 198 dyes. J Water Reuse Desalin.

[56] Atmani F, Kaci MM, Yeddou-Mezenner N, Soukeur A, Akkari I, Navio JA (2022). Insights into the physicochemical properties of Sugar Scum as a sustainable biosorbent derived from sugar refinery waste for efficient cationic dye removal. Biomass Convers Biorefin.

[57] Kumar A, Jena HM (2016). Preparation and characterization of high surface area activated carbon from Fox nut (Euryale ferox) shell by chemical activation with H_3_PO_4_. Results Phys.

[58] Yorgun S, Yildiz D (2015). Preparation and characterization of activated carbons from Paulownia wood by chemical activation with H_3_PO_4_. J Taiwan Inst Chem Eng.

[59] Tiryaki B, Yagmur E, Banford A, Aktas Z (2014). Comparison of activated carbon produced from natural biomass and equivalent chemical compositions. J Anal Appl Pyrolysis.

[60] Zhou L, Li M, Sun Y, Zhou Y (2001). Effect of moisture in microporous activated carbon on the adsorption of methane. Carbon.

[61] Kiliç M, Apaydin-Varol E, Pütün AE (2012). Preparation and surface characterization of activated carbons from *Euphorbia rigida* by chemical activation with ZnCl_2_, K_2_CO_3_, NaOH and H_3_PO_4_. Appl Surf Sci.

[62] Queiroz LS (2020). Activated carbon obtained from amazonian biomass tailings (acai seed): modification, characterization, and use for removal of metal ions from water. J Environ Manag.

[63] Chandra TC, Mirna MM, Sunarso J, Sudaryanto Y, Ismadji S (2009). Activated carbon from durian shell: preparation and characterization. J Taiwan Inst Chem Eng.

[64] Maulina S, Iriansyah M. Characteristics of activated carbon resulted from pyrolysis of the oil palm fronds powder. In: IOP conference series: materials science and engineering. 2018;309(1):012072. 10.1088/1757-899X/309/1/012072.

[65] Verma R, Kundu LM, Pandey LM (2021). Enhanced melanoidin removal by amine-modified *Phyllanthus emblica* leaf powder. Bioresour Technol.

[66] Zhou Y (2021). Modulating hierarchically microporous biochar via molten alkali treatment for efficient adsorption removal of perfluorinated carboxylic acids from wastewater. Sci Total Environ.

[67] Prakash MO, Raghavendra G, Ojha S, Panchal M (2021). Characterization of porous activated carbon prepared from arhar stalks by single step chemical activation method. Mater Today Proc.

[68] Mopoung S, Dejang N (2021). Activated carbon preparation from eucalyptus wood chips using continuous carbonization–steam activation process in a batch intermittent rotary kiln. Sci Rep.

[69] Mondal S, Aikat K, Halder G (2016). Biosorptive uptake of ibuprofen by chemically modified *Parthenium hysterophorus* derived biochar: equilibrium, kinetics, thermodynamics and modeling. Ecol Eng.

[70] Kalavathy H, Regupathi I, Pillai MG, Miranda LR (2009). Modelling, analysis and optimization of adsorption parameters for H_3_PO_4_ activated rubber wood sawdust using response surface methodology (RSM). Colloids Surf B Biointerfaces.

[71] Chowdhury S, Das Saha P (2012). Scale-up of a dye adsorption process using chemically modified rice husk: optimization using response surface methodology. Desalin Water Treat.

[72] Fito J (2023). Adsorption of methylene blue from textile industrial wastewater using activated carbon developed from *Rumex abyssinicus* plant. Sci Rep.

[73] Jain M, Garg VK, Kadirvelu K (2011). Investigation of Cr(VI) adsorption onto chemically treated *Helianthus annuus*: optimization using response surface methodology. Bioresour Technol.

[74] Bello OS, Adegoke KA, Sarumi OO, Lameed OS (2019). Functionalized locust bean pod (*Parkia biglobosa*) activated carbon for Rhodamine B dye removal. Heliyon.

[75] Hii HT (2021). Adsorption isotherm and kinetic models for removal of methyl orange and Remazol Brilliant Blue R by coconut shell activated carbon. Trop Aquat Soil Pollut.

[76] Ozturk G, Silah H (2020). Adsorptive removal of Remazol Brilliant Blue R from water by using a macroporous polystyrene resin: isotherm and kinetic studies. Environ Process.

[77] Li W, Sun H, Yapaoz MA, Attar A. Equilibrium and thermodynamic studies of adsorption of Remazol Brilliant Blue dye on snail shell powder. In: IOP conference series: materials science and engineering; 2018. 10.1088/1757-899X/871/1/012036.

[78] Thiam A (2020). Valorization of date pits as an effective biosorbent for Remazol Brilliant Blue adsorption from aqueous solution. J Chem.

[79] Ahmad M, Eusoff MA, Oladoye PO, Adegoke KA, Bello OS (2020). Statistical optimization of Remazol Brilliant Blue R dye adsorption onto activated carbon prepared from pomegranate fruit peel. Chem Data Collect.

